# Gene-expression reversal of lncRNAs and associated mRNAs expression in active vs latent HIV infection

**DOI:** 10.1038/srep34862

**Published:** 2016-10-19

**Authors:** Madhavan Nair, Vidya Sagar, Sudheesh Pilakka-Kanthikeel

**Affiliations:** 1 Department of Immunology, Herbert Wertheim College of Medicine, Florida International University, Miami, FL-33199, USA; 2 Institute of Neuro-Immune Pharmacology, Herbert Wertheim College of Medicine, Florida International University, Miami, FL-33199, USA; 3 Center for Personalized Nanomedicine, Herbert Wertheim College of Medicine, Florida International University, Miami, FL-33199, USA

## Abstract

Interplay between lncRNAs and mRNAs is rapidly emerging as a key epigenetic mechanism in controlling various cell functions. HIV can actively infect and/or can persist latently for years by manipulating host epigenetics; however, its molecular essence remains undiscovered in entirety. Here for the first time, we delineate the influence of HIV on global lncRNAs expression in monocytic cells lines. Our analysis revealed the expression modulation of nearly 1060 such lncRNAs which are associated with differentially expressed mRNAs in active and latent infection. This suggests a greater role of lncRNAs in regulating transcriptional and post-transcriptional gene expression during HIV infection. The differentially expressed mRNAs were involved in several different biological pathways where immunological networks were most enriched. Importantly, we discovered that HIV induces expression reversal of more than 150 lncRNAs between its active and latent infection. Also, hundreds of unique lncRNAs were identified in both infection conditions. The pathology specific “gene-expression reversal” and “on-and-off” switching of lncRNAs and associated mRNAs may lead to establish the relationship between active and HIV infection.

The highly active antiretroviral therapy (HAART) can certainly decrease the plasma viral load below the detection limit; however, complete elimination of HIV infection remains far out of reach. The major hindrance in this direction is the latent HIV infection in cell subpopulations where anti-HIV drugs and immune clearance is not effective. HIV can persist in these viral reservoirs for years with little or no productive infection and can reactivate later^1^. In contrast to initial belief, latent HIV reservoirs are present beyond the realm of memory CD4^+^ T cells^2^,^3^,^4^,^5^. Subpopulations of all kind of immune cells such as dendritic cells, hematopoietic progenitor cells, natural killer cells, mast cells, monocytes, macrophages, etc. also serve as HIV reservoirs. It is noteworthy to mention that infected/non-infected cells of monocyte-macrophage lineage naturally transmigrate to brain from peripheral circulation and vice-versa. This results in HIV dissemination into brain. CNS is also reported to be a major site of HIV reservoir^2^,^6^. Thus, macrophages/monocytes serve as major connecting link between peripheral and brain infection of HIV, be it active or latent^7^. It has been widely accepted that HIV latency is driven by multitude of epigenetic-based transcriptional factors after integration of pro-viral genomes into the host genome^8^,^9^,^10^. Role of histone modifications such as acetylation, methylation, etc. in HIV latency have already been shown in several studies^11^,^12^,^13^,^14^. However, these factors proved not more than a fractional player and despite intensive efforts by global scientific community during last decade, the molecular essence of HIV latency still remains indistinct.

The epigenetic landscape - beyond histone modifications - is extended deep into the genomic “dark matter”. This refers to non-protein-coding RNAs which comprises nearly 70% of genome. These are categorized into small and long non-coding RNAs (lncRNAs) with later being >200nt in length^15^. Similarly, plethora of research suggest possible role of one or more microRNAs (miRNAs) in the HIV latency^16^. While vast majority of small non-coding RNAs (e.g. miRNAs) are being researched over a decade, functional role of lncRNAs has commenced only recently. Human genome is expected to have more than 23,000 lncRNAs that can regulate various transcriptional and posttranscriptional processes^17^. The complex paradigm of lncRNAs as epigenetic modulators is fast being revealed. Many lncRNAs signatures display intimate connection with histone proteins associated epigenetic marks. These can tune the chromatin activation/repression and chromosomal looping^18^. Also, lncRNAs can guide gene-silencing via targeted recruitment of epigenetic silencing complexes in the promoter region and suppression of lncRNAs has been shown to activate their targeted protein-coding gene^19^. The lncRNAs can function both, *in trans* and *in cis i.e.* when their targeted genes are located on different and same allele, respectively^15^. Thus, as an endogenous effector molecule, lncRNAs show remarkable influence on epigenetic regulations with a surprising degree of complexity.

Most pathogens are adept at altering host gene expression in their favor by controlling replication, transcription, and/or translation processes. As such, modulation of lncRNAs – which is emerging as a vital player at transcriptional and post-transcriptional level – may be a critical point of manipulation by obligate parasites such as HIV. In fact, few existing reports at this point suggests role of lncRNAs in activation of HIV replication and subsequent increase in virus production as well^20^,^21^,^22^. This provides a clue that defining lncRNAs contribution in HIV biogenesis may represent tools needed to beat the enigma behind latent and active infection. To best of our knowledge no studies, to-date, have been conducted to delineate variations in the lncRNAs profile between an active and latent HIV infection. Using microarray analysis we hereby, for the first time, report that active and latent HIV infection results in differential pattern of lncRNAs and mRNAs expression. Gene ontology and pathways analysis of upregulated and downregulated transcripts showed their relevance to several metabolic and biological processes. While both, active and latent infection shared common upregulated and downregulated lncRNAs and mRNAs in compare to uninfected control, majority of them were uniquely expressed in both infections scenario. Importantly, a big chunk of these lncRNAs and mRNAs showed expression reversal when compared between active and latent infection and vice-versa. Also, more than one thousands of both, lncRNAs and their associated mRNAs were differentially expressed in active and/or latent infection. As such, this study may lead to decode illusionary linkage driving the HIV latency, which continues to be the biggest stumbling block in developing successful antiretroviral drug cocktails.

## Materials and Methods

### HIV infection and RNA isolation

The U-937 monocytes (American Type Culture Collection) and promonocytic U1 latent cells (AIDS Research and Reference Reagent Program, NIAID, National Institute of Health, Rockville, MD, catalog no. 165) were used to perform active and latent infection model, respectively. U1 is a subclone of U937 that has been chronically infected with HIV-1, and show minimal constitutive expression of virus. The U-937 monocytes were infected with 100 ng/ml HIV-1_Ba-L_(NIH AIDS Research and Reference Reagent Program catalog no. 510). After 5 days of infection, RNA were isolated using the RNeasy mini kit (Qiagen, Limburg, the Netherlands) according to the manufacturer’s guidelines.

### LncRNAs Microarray analysis

For lncRNAs microarray analysis, Agilent Array platform was employed. The sample preparation and microarray hybridization were performed based on the manufacturer’s standard protocols with minor modifications. Briefly, mRNA was purified from total RNA after removal of rRNA (mRNA-ONLY™ Eukaryotic mRNA Isolation Kit, Epicentre). Then, each sample was amplified and transcribed into fluorescent cRNA along the entire length of the transcripts without 3′ bias utilizing a mixture of oligo(dT) and random primers (Arraystar Flash RNA Labeling Kit, Arraystar). The labeled cRNAs were hybridized onto the Human LncRNA Array v3.0 (8 × 60 K, Arraystar). Arraystar Human LncRNA Microarray V3.0 is designed for the global profiling of about 30,586 human LncRNAs. The LncRNAs are carefully constructed using the most highly-respected public transcriptome databases (Refseq, UCSC knowngenes, Gencode, etc), as well as landmark publications. Each transcript is represented by a specific exon or splice junction probe which can identify individual transcript accurately. Positive probes for housekeeping genes and negative probes are also printed onto the array for hybridization quality control. After having washed the slides, the arrays were scanned by the Agilent Scanner G2505C. Agilent Feature Extraction software (version 11.0.1.1) was used to analyze acquired array images. Quantile normalization and subsequent data processing were performed using the GeneSpring GX v12.1 software package (Agilent Technologies). After quantile normalization of the raw data, LncRNAs that at least 4 out of 12 samples have flags in Present or Marginal were chosen for differentially expressed LncRNAs screening.

### Analysis of mRNAs microarray

Sample preparation, RNA labeling, and array hybridization were similar to that explained for lncRNAs microarray in above section. Arraystar Human Microarray V3.0 is designed for the global profiling of about 26,109 coding transcripts. After quantile normalization of the raw data, mRNAs that at least 4 out of 12 samples have flags in Present or Marginal was chosen for differentially expressed mRNAs screening. The GO categories for differentially expressed mRNAs were derived from Gene Ontology (http://www.geneontology.org), which comprise three structured networks of defined terms that describe gene product attributes. The pathway analysis for differentially expressed mRNAs was based on the latest KEGG (Kyoto Encyclopedia of Genes and Genomes, (http://www.genome.jp/kegg) database.

### Statistics

Quality assessment of LncRNAs and mRNAs data after filtering was performed using box-plot. The Box Plot was used for comparing the distributions of the intensities from all samples. After normalization, the distributions of log2-ratios among all tested samples were nearly the same. For screening differentially expression, lncRNAs and mRNAs that passed Volcano Plot filtering (Fold Change >= 2.0 and P-value <= 0.05) was considered statistically significance. The P-value cut-off for both GO term and pathways is 0.05.

## Results

### Uniquely modulated and shared lncRNAs between active vs latent HIV infection

**Figure 1:**
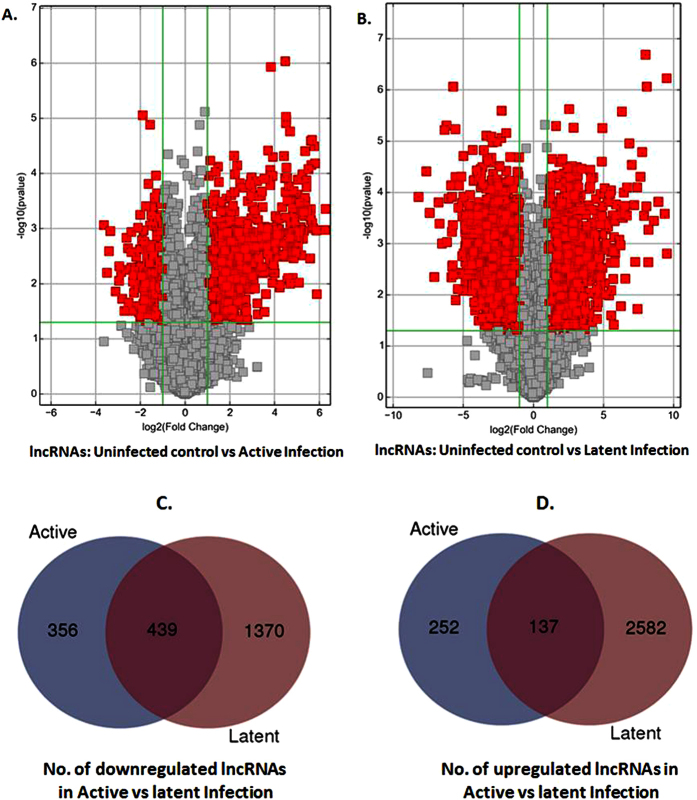
(**A,B**) Volcano plots of the two samples t-tests of lncRNAs expression fold change for active (**A**) and latent (**B**) HIV infections in macrophages in compare to uninfected control: It shows differentially expressed lncRNAs with statistical significance that passed Volcano Plot filtering (Fold Change >= 2.0 and P-value <= 0.05). The vertical yellow lines correspond to 2.0-fold up and down and the horizontal yellow line represents a P-value up to 0.05. So the red cubes in the plot represent the differentially expressed lncRNAs with statistical significance. (**C,D**) Venn diagram showing no of common downregulated (**C**) and upregulated (**D**) lncRNAs in active vs latent infection.

To gain an understanding of epigenetics complexity of HIV infection, active and latently infected macrophages were subjected for lncRNAs expression profiling. Figure 1A,B shows the volcano plots for the differential expression of lncRNAs in uninfected vs active infection and uninfected vs latent infection respectively. Based on microarray analysis, 795 lncRNAs were found downregulated (Fig. 1C) and 389 lncRNAs were found upregulated (Fig. 1D) during the active infection, when compared to uninfected control. In case of latent infection, no. of downregulated and upregulated lncRNAs were 1809 (Fig. 1C) and 2719 (Fig. 1D), respectively. Inclusion of upregulated or downregulated lncRNAs was based on at least 2 fold modulation of their gene expression with p-value of 0.05. A total of 439 downregulated (Fig. 1C) and 137 upregulated (Fig. 1D) lncRNAs were found common in both active and latent infection. As such, several lncRNAs were specifically downregulated and upregulated in active and latent infection. During active infection, 356 (Fig. 1C) and 252 (Fig. 1D) lncRNAs were uniquely downregulated and upregulated, respectively. Similarly, 1370 (Fig. 1C) and 2582 (Fig. 1D) lncRNAs were respectively downregulated and upregulated during latent infection.

### Uniquely modulated and shared mRNAs between active vs latent HIV infection

**Figure 2:**
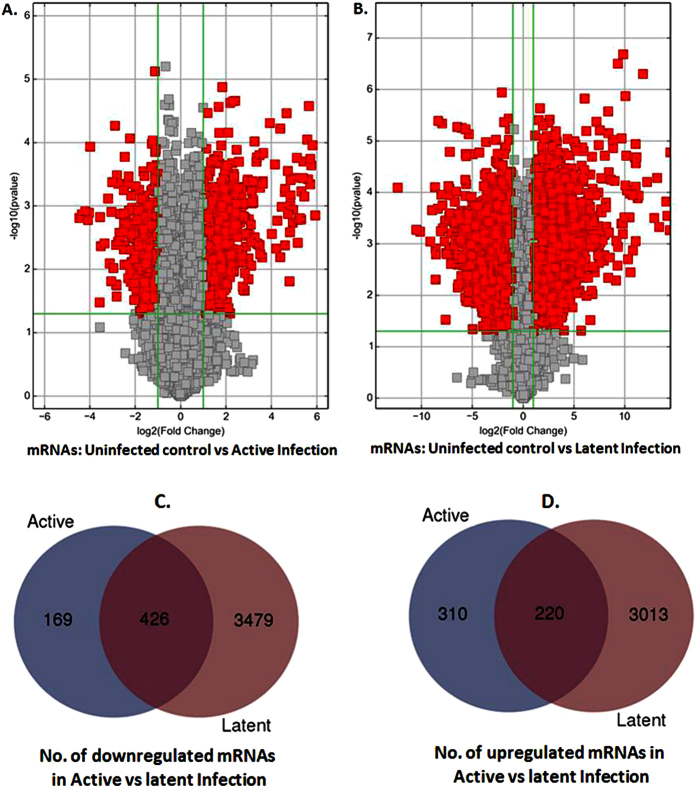
(**A,B**) Volcano plots of the two samples t-tests of mRNAs expression fold change for active (**A**) and latent (**B**) HIV infections in macrophages in compare to uninfected control: It shows differentially expressed lncRNAs with statistical significance that passed Volcano Plot filtering (Fold Change >= 2.0 and P-value <= 0.05). The vertical yellow lines correspond to 2.0-fold up and down and the horizontal yellow line represents a P-value up to 0.05. So the red cubes in the plot represent the differentially expressed mRNAs with statistical significance. (**C,D**) Venn diagram showing no of common downregulated (**C**) and upregulated (**D**) lncRNAs in active vs latent infection.

Similar to lncRNAs expression pattern, several protein-coding gene expression modulations were specific to either latent or active infections and many upregulated and downregulated transcripts were common to both conditions as well. Figure 2A,B represents the volcano plots for the differential expression of mRNAs in uninfected vs active infection and uninfected vs latent infection respectively. In comparison to uninfected control, active infections resulted in downregulation of 595 mRNAs (Fig. 2C) and upregulation of 530 mRNAs (Fig. 2D). The latent infection resulted in downregulation of 3905 mRNAs (Fig. 2C) and upregulation of 3233 mRNAs (Fig. 2D). Again, inclusion of upregulated or downregulated mRNAs was based on their higher or lower expression by at least 2 fold and having p-value of 0.05. Off these modulated protein-coding genes, 426 downregulated (Fig. 2C) and 220 (Fig. 2D) upregulated mRNAs were common to both active and latent infections. As such, 169 downregulated mRNAs (Fig. 2C) and 310 upregulated mRNAs (Fig. 2D) were specific to active infection and 3479 downregulated mRNAs (Fig. 2C) and 3013 upregulated mRNAs (Fig. 2D) were specific to latent condition. Details of all modulated mRNAs have been given in supplementary data 2.

**Figure 3:**
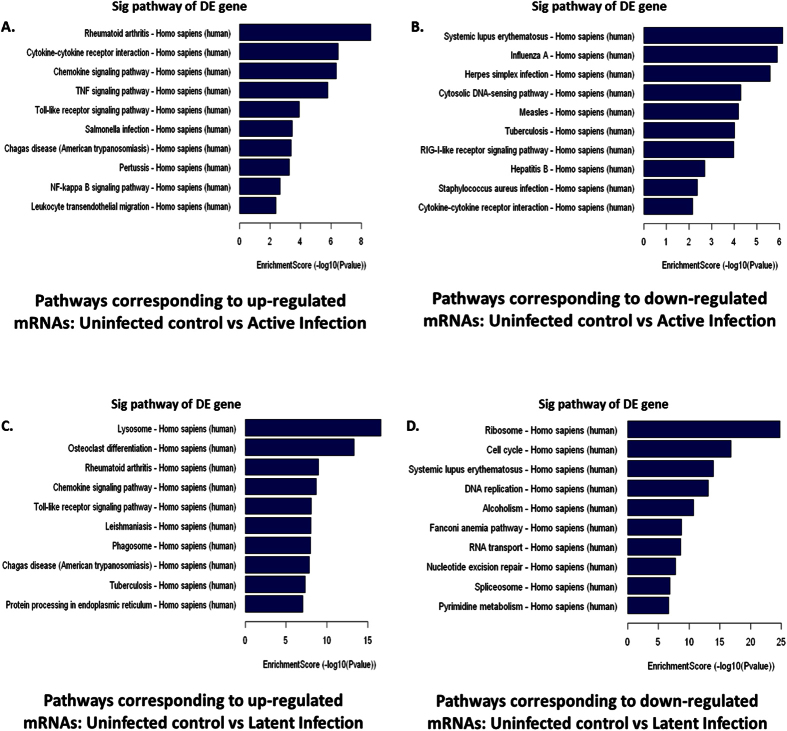
Pathway analyses of differentially expressed mRNAs were based on latest KEGG (Kyoto Encyclopedia of Genes and Genomes, http://www.genome.jp/kegg) database. Most enriched pathway of upregulated (**A**) and downregulated (**B**) mRNAs in active infection condition was dominated by immunological networks. Pathway corresponding to upregulated mRNAs in latent infection (**C**) also includes many immunological networks and pathway corresponding to downregulated mRNAs in latent infection (**D**) showed enrichment of replication associated pathways. These differentially expressed mRNAs are associated with 155 biological pathways (Supplementary figure 2), where nodes with red color denotes significant association.

Differentially expressed mRNAs belonging to both active and latent infection were subjected to GO (Supplementary figures 1) and pathway analysis (Fig. 3). GO analysis of transcripts was annotated under three ontological categories, namely, biological process, cellular component, and molecular function. The significance of GO term enrichment in the differentially expressed transcripts was estimated based on p values ≤ 0.05. As expected, different “most highly enriched GO terms” for active and latent infection were found (Supplementary figures 1A-D) because – despite numbers of common upregulated and downregulated mRNAs between active and latent infection – large fraction was specific to either infection condition (Figs 1C,D and 2C,D). Details of each GO term are given in supplementary data 3 and the GO associations of each upregulated and downregulated transcript are given in supplementary data 2.

Moreover, pathways analysis showed that the modulated genes are involved in 155 different pathways ((data not included). This analysis allows determining the biological pathway in which there is a significant enrichment of differentially expressed mRNAs; and again the p-value ≤ 0.05 denotes the significance of the Pathway (Fig. 3). Again, active and latent infection showed different “most enriched network” (Fig. 3A–D). Immunological networks such as cytokine-cytokine receptor interaction, chemokine signaling pathway, TNF signaling pathway, Toll-like receptor signaling pathway, Rheumatoid arthritis pathway, NF-kappa B singling pathway, and leukocyte transendothelial migration pathway were found among most enriched network in active infection corresponding to upregulated genes (Fig. 3A). The pathway corresponding to downregulated mRNAs during active condition also included inflammatory pathways such as Rig-I-like receptor signaling pathway, cytokine-cytokine receptor interaction, lupus erythematosus, and cytosolic DNA-sending pathway (Fig. 3B). In the latent infection corresponding to upregulated mRNAs, immunological pathways such as lysosome pathway, chemokine signaling pathway, Toll-like receptor signaling pathway, Phagosome pathway, etc. were enriched (Fig. 3C). Interestingly, downregulated genes in latent infection showed enrichment of replication associated pathways such as, DNA replication pathway, RNA transport pathway, Nucleotide excision pathway, Spliceosome pathway, and Pyrimidine metabolism (Fig. 3D). Some HIV associated secondary infection pathway, such as, salmonella infection, influenza infection, herpes simplex virus infection, tuberculosis, pertussis, hepatitis B, staphylococcus infection, etc. are also enriched across both active and latent infection.

### Expression reversal of lncRNAs and mRNAs between active vs latent HIV infection

**Figure 4:**
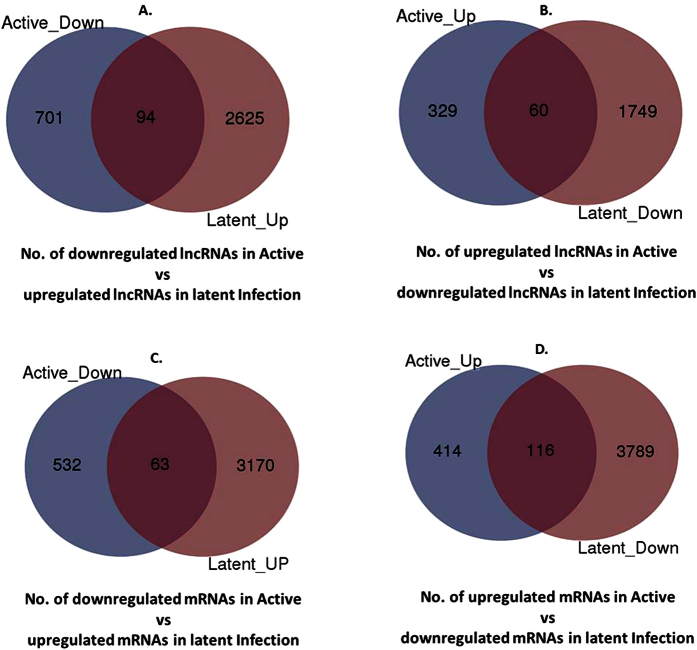
Gene expression reversal: Venn analysis showing number of those lncRNAs (**A,B**) and mRNAs (**C,D**) which were both downregulated and upregulated in active vs latent infection and vice versa. It was found that 94 lncRNAs downregulated in active infection were also upregulated in latent infection (**A**) and 60 lncRNAs upregulated in active infection were also downregulated in latent infection (**B**). Similarly, 63 mRNAs downregulated in active infection were also upregulated in latent infection and 116 mRNAs upregulated in active infection were also downregulated in latent infection. Details of these lncRNAs and mRNAs are given in supplementary data 1 and 2, respectively.

We wanted to examine if HIV induces certain gene expression during active infection which are suppressed in latent infection or vice-versa. Interestingly, the gene expression reversal between active and latent infections was found at both lncRNAs and mRNAs level (Fig. 4). While 94 lncRNAs upregulated in latent infection were downregulated in active infection (Fig. 4A), 60 downregulated lncRNAs in latent infection were upregulated in active infection (Fig. 4B). As such, a total of 154 lncRNAs showed expression reversal between both infection conditions. Similarly 179 mRNAs were expressed inversely in active and latent infection: 63 downregulated 116 upregulated mRNAs in active infection were respectively downregulated and upregulated in latent infection (Fig. 4C,D). Details of lncRNAs and mRNAs showing expression reversal have been given in supplementary data 4.

### Association between differentially expressed lncRNAs and mRNAs

**Figure 5:**
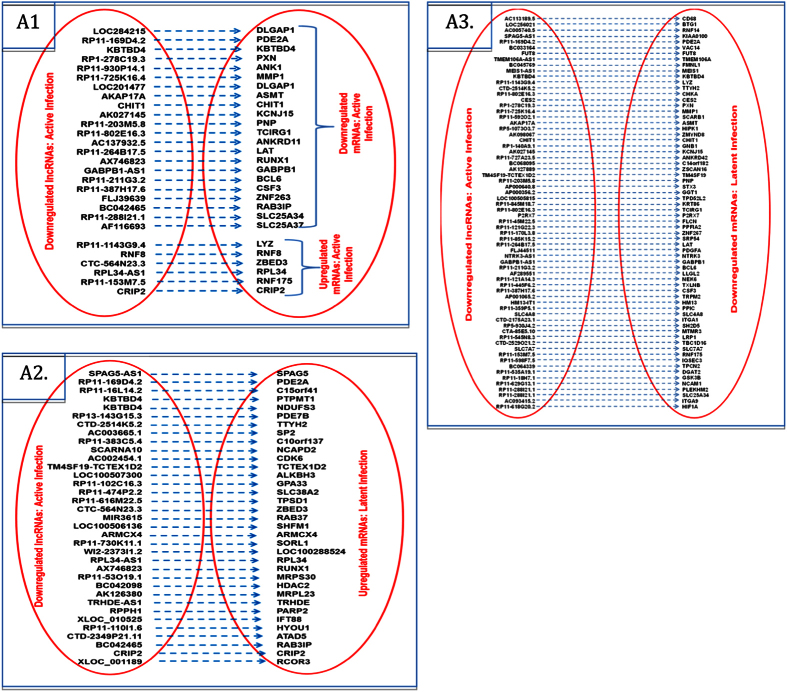
Interplay between differentially expressed lncRNAs and their associated mRNAs: **A1**: Pairs of lncRNAs downregulated in active infection and mRNAs downregulated and upregulated in active infection; **A2**: Pairs of lncRNAs downregulated in active infection and mRNAs upregulated in latent infection; **A3**: Pairs of lncRNAs downregulated in active infection and mRNAs downregulated in latent infection.

**Figure 6:**
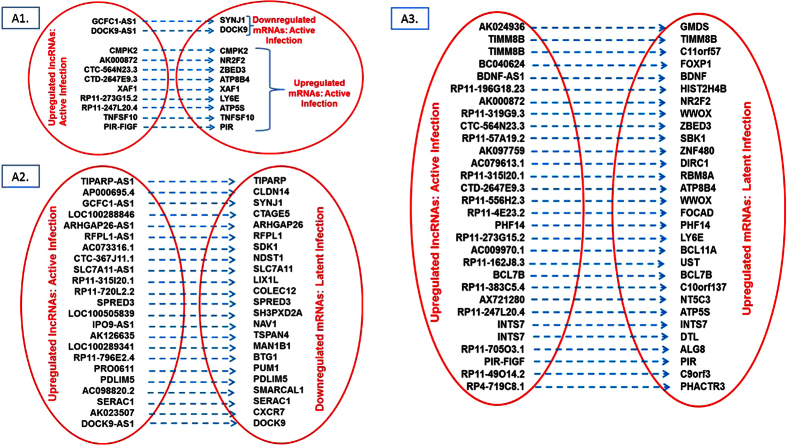
A1: Pairs of lncRNAs upregulated in active infection and mRNAs downregulated and upregulated in active infection; A2: Pairs of lncRNAs upregulated in active infection and mRNAs downregulated in latent infection. **A3**: Pair of lncRNAs upregulated in active infection and mRNAs upregulated in latent infection.

**Figure 7:**
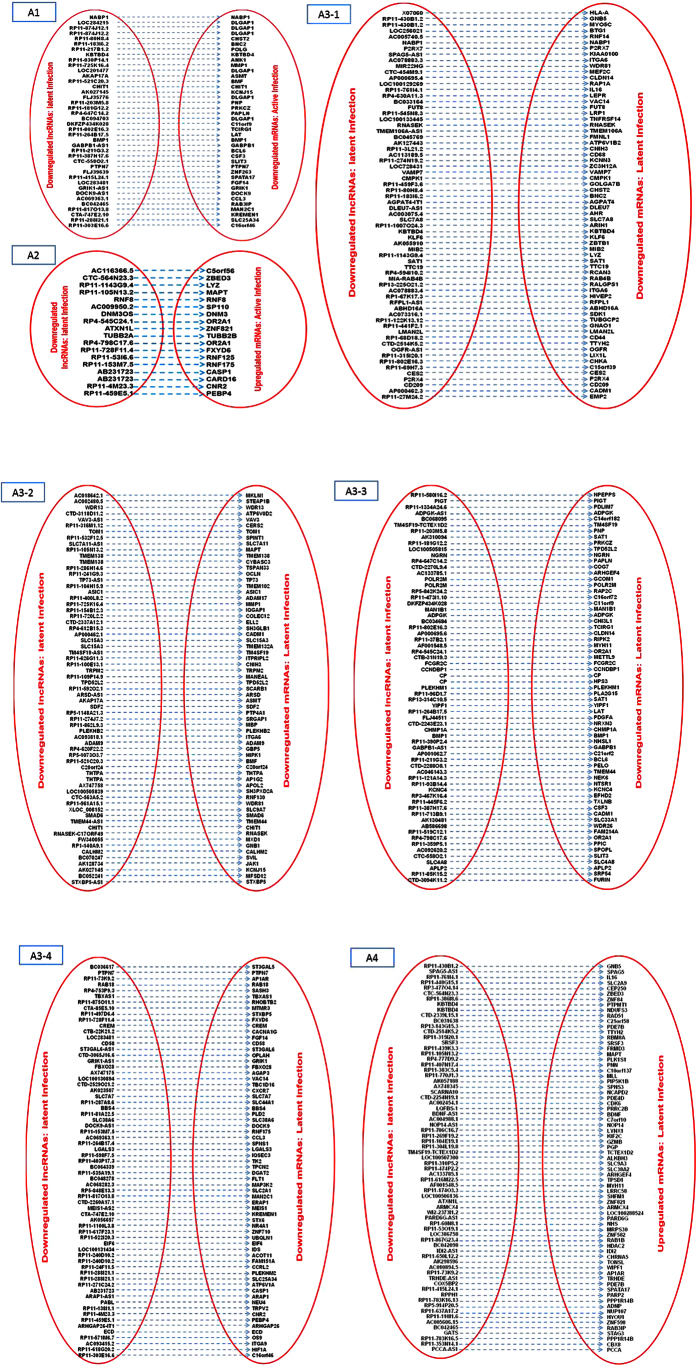
**A1**: Pairs of lncRNAs downregulated in latent infection and mRNAs downregulated in active infection; **A2**: Pairs of lncRNAs downregulated in latent infection and mRNAs upregulated in active infection; **A3**: 1–4: Pairs of lncRNAs downregulated in latent infection and mRNAs downregulated in latent infection; A4: Pairs of lncRNAs downregulated in latent infection and mRNAs upregulated in latent infection.

**Figure 8:**
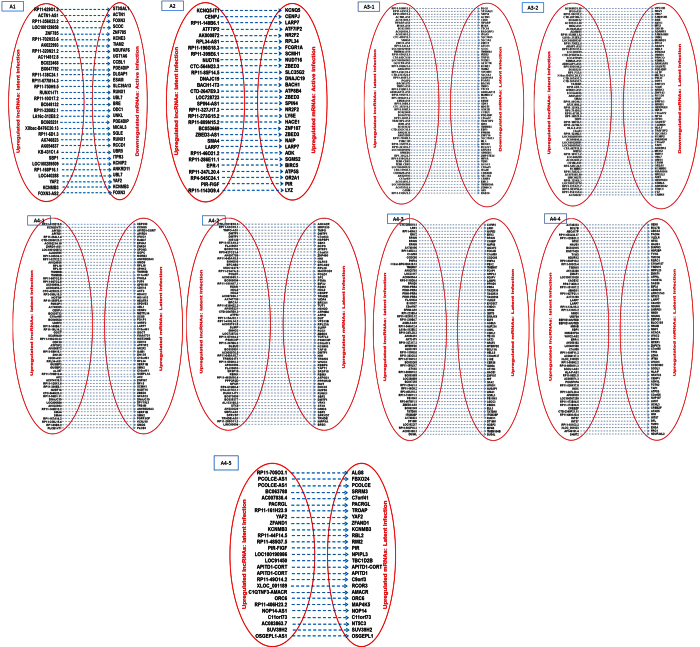
**A1**: Pairs of lncRNAs upregulated in latent infection and mRNAs downregulated in active infection; **A2**: Pairs of lncRNAs upregulated in latent infection and mRNAs upregulated in active infection; **A3**: 1–2: Pairs of lncRNAs upregulated in latent infection and mRNAs downregulated in latent infection; **A4**: 1–5: Pairs of lncRNAs upregulated in latent infection and mRNAs upregulated in latent infection.

To establish the pattern of interplay between lncRNAs and their associated mRNAs, we analyzed if both RNAs category were differentially expressed in active and/or latent infection. We found 1068 such pair with parallel (lncRNAs upregulation/downregualtion => mRNAs upregulation/downregulation) and/or reverse expression (lncRNAs upregulation/downregualtion => mRNAs downregulation/upregulation) pattern. All differentially expressed “lncRNAs – mRNAs” pair is denoted in Figs 5, 6, 7 and 8. While role of these lncRNAs remain undiscovered, several mRNAs from such pair have been implicated in HIV infection.

## Discussion

HIV latency is a state of replication restriction when either few viruses are reproduced or viruses are reproduced intermittently. The quantitative and/or temporal dimension of establishment of HIV persistence is determined by many factors such as cell’s life span, their infection susceptibility and proliferative ability, alterations in cellular physiology and/or immunologic controls, etc. Cells of monocyte/macrophages lineage is one of the principal targets of HIV among others. These cells are more resistant - than CD4^+^ T or other cells - to the cytopathic effect induced via apoptosis during HIV infection. In turn, they make more stable hideouts and can harbor HIV for longer time. As an important viral reservoir, macrophages can disseminate HIV across all organ systems because of its universal presence in body^23^. A decade of efforts made in the direction of eliminating HIV reservoirs is strongly suggesting towards involvement of unknown, sophisticated epigenetic regulatory machinery that is beyond known landscape of histone modifications and small non-coding RNAs such as miRNAs. The complex paradigm of lncRNAs-based transcriptional and post-transcriptional gene regulation is gradually being unfolded and evidences insinuate them as a central player in driving epigenetic remodeling in different non-viral^24^ and viral disease systems such as SARS corona virus, influenza virus, Marek’s disease virus, herpes simplex virus, etc.^17^. Couple of recent studies shows specific lncRNA-based modulation of HIV replication. Nonetheless, lncRNAs in these studies were selected based on their role in other diseases^21^,^22^. Another study by Saayman *et al.*^24^ examined HIV-encoded antisense lncRNAs in epigenetic control of HIV transcriptions. Discovery of new, physiologically relevant lncRNAs continues to rise in human cells and as such examining one or few dozen lncRNAs from other disorders may little reflect the cumulative intensities about their role in HIV infection. In this microarray analysis, we profiled global lncRNAs in both active and latent infection. With a profiling ability of 30,586 human LncRNAs, our assay could detect 1184 differentially expressed lncRNAs in active infection and 4523 differentially expressed lncRNAs in latent infection. This difference in the no. of lncRNAs between active and latent infection may be attributed to the differences in the degree or intensity of epigenetic modifications during respective infection stages. The continued transcriptional suppression of HIV genome post-integration, for latency maintenance, may require greater degree of epigenomic involvement compared to active infection where epigenomic alterations may be of little significance. Moreover, intrinsic transcriptional originality of the cell lines may also partially contribute to these differences because promonocytic U1 latent cells, a subclone of U937, are chronically infected with HIV-1 compared to U-937 monocytes used for active HIV-1 infection. Many of these lncRNAs were specifically modulated during active or latent infection. Nonetheless a big amount of them were either upregulated or downregulated in both infection scenarios. These variations in lncRNAs expression between two different HIV infection conditions further substantiate the greater role of epigenetic regulators and that lncRNAs may have specific “on-and-off” switching mechanism to drive latent or active condition. Understanding such mechanism – if exists – can better be revealed by knowing their chromosomal locations and its relationship with nearby coding gene. We found that these modulated lncRNAs during HIV active or latent infection are distributed on both “+” and “−” strand of different chromosomes and belongs to all groups: intergenic, intron sense-overlapping, exon-sense overlapping, natural antisense, intronic antisense, and bidirectional (supplementary data 1). This knowledge can certainly be helpful in determining lncRNAs associated host factors responsible for guiding epigenetic remodeling.

A more holistic understanding of lncRNAs mechanism requires establishing their association with mRNAs. Several studies have shown that lncRNAs can behave as transcription enhancer or suppressor in an orientation-independent or dependent manner^25^. As such, we first established global profiling of mRNAs in both active and latent infection. With a profiling ability of 26,109 coding transcripts, we found 1125 and 7138 mRNAs differentially modulated during active and latent infection, respectively. Similar to lncRNAs, modulated mRNAs were specific to and shared by active and latent infection. This quantitative pattern of mRNA modulations between active and latent infection is in parallel with that of lncRNAs suggesting high degree of lncRNAs involvement in epigenetic modifications and subsequent transcriptional control. Importantly, this raises the possibility that HIV may induce parallel manipulation of lncRNAs and mRNAs as a mechanism to dominate host cellular machinery. Nonetheless, this parallel manipulation may exist for both unrelated or closely associated lncRNAs and mRNAs. We could establish about 1068 lncRNAs and associated mRNAs pair modulated during HIV active and/or latent infection. Role of lncRNAs in regulation of marker genes have been established in other diseases^25^. In fact, several of mRNAs from the modulated “lncRNAs-mRNAs” pair have been established in the HIV infection. For example, Histone deacetylase (HDAC) regulates transcription of numerous cellular and viral genes. It has been shown that the class I HDACs, HDAC1, −2, and −3, are recruited to the HIV-1 LTR in cell line models of HIV-1 latency^26^. The sub-class I selective inhibitors for HDAC1, −2, and −3 have shown to induce latent HIV-1 expression strongly in resting CD4 + T cells^27^. Moreover, tat induced upregulation of HDAC2 are also reported to induce transcriptional repression leading to neurocognitive impairment^28^. The RUNX protein, which works in combination with the co-factor CBF-β, function as critical transcriptional regulators in T-cells^29^. RUNX1 bind DNA sequences within the HIV-1 LTR repressing the transcription. Lower expression of BDNF has been reported in HIV-infected individuals with HAD than in non-demented HIV^30^. The decrease in BDNF evoked by HIV is believed to contribute to the development of synaptic simplification and neuronal damage seen in HAD. BRCA1, which is present at the HIV-1 LTR in a highly relevant model of HIV infection, is another protein found to be important for viral transcription. The cells that lack BRCA1 have severely reduced HIV-1 Tat-dependent transcription^31^. Gain or loss-of-function studies have shown to enhance or decrease transcription. Moreover, BRCA1 has been shown to be in complex with pTEF-b, and Cyclin T1 has been shown to be an essential factor for BRCA1-dependent activation of RNAP II transcription.

Similarly, other modulated mRNAs also regulates HIV infection in one or other way. In fact, pathways and gene ontology analysis shows their involvement in 155 biological cycles where majority of enriched network belonged to immunological and inflammatory significance. This was expected because most infection including that by HIV induces immunological and inflammatory defense signaling. HIV infection induces various secondary infections and therefore enrichment of network associated with salmonella infection, influenza infection, herpes simplex virus infection, tuberculosis, pertussis, hepatitis B, staphylococcus infection, etc. may imply the likelihood of such opportunistic infections during HIV pathogenesis. Interestingly, enrichment of replication associated pathways (such as RNA transport pathway, Nucleotide excision pathway, Spliceosome pathway, and Pyrimidine metabolism, etc.) during latent infection suggest towards a highly sophisticated and regressive epigenetics overhauling in compare to active infection. This is also reflected in the quantitative differences of the total differentially expressed lncRNAs and mRNAs between active and latent infection. Now challenge ahead is to examine pathophysiological significant of these closely associated, differential expressed “lncRNAs-mRNAs” pair one by one. Nonetheless, active and latent stages are two opposite facet of the HIV infection and therefore, expression reversal of a specific gene set (Active upregulation/downregualtion <=> latent downregulation/upregulation) between these two conditions cannot be excluded. It is possible that expression reversal of certain genes between active and latent infection, independently or together with the active and latent infection-specific uniquely modulated genes, can have remarkable pathophysiological significance. Genetic commonality (close association) may potentiate susceptibility of other related disorders. In fact, copy number variants and temporal expression differences of a specific gene associated with one neurological and autoimmune disorder can predispose to others^32^. Thus, driver of latent infection of HIV may find its epigenetic origin in active infection.

A correlation at the interface of two different pathological condition of same disease can be drawn with the discovery of multiple epigenetic signatures. Disrupted expression of even a single coding or non-coding gene possesses ability to affect multiple molecular pathways and as such, different pathological phenotypes may entangle in converged molecular circuits. Interpretation of underlying disease mechanism(s) based on single gene annotation often proved to be less comprehensive^33^. Thus, discoveries of a set of unique or shared dysregulated molecular markers between active and latent HIV infection can accelerate studies on their pathophysiologic mechanisms and in turn can lead to disease-modifying therapy. Nonetheless, defining cell-specific epigenetic modulations can only represent a small subset when their arrangements are also dictated on the basis of neighboring cell connectivity, morphology, and physiological properties^34^. Exploratory follow-up of our macrophage specific findings - involving clinical resolution – will substantiate the lncRNAs-mRNAs functional similarities or dissimilarities between active and latent infections.

## Additional Information

**How to cite this article**: Nair, M. *et al.* Gene-expression reversal of lncRNAs and associated mRNAs expression in active vs latent HIV infection. *Sci. Rep.* **6**, 34862; doi: 10.1038/srep34862 (2016).

## Supplementary Material

Supplementary Information

Supplementary Data 1

Supplementary Data 2

Supplementary Data 3

Supplementary Data 4
