# Supplementary material for: Gene-expression reversal of lncRNAs and associated mRNAs expression in active vs latent HIV infection
Source: Sci Rep. 2016 Oct 19;6:34862. doi: 10.1038/srep34862 (PMC5069461; doi:10.1038/srep34862)

Gene-expression reversal of lncRNAs and associated mRNAs expression in active vs latent HIV infection
Madhavan Nair, Vidya Sagar and Sudheesh Pilakka-Kanthikeel

Supplementary Figure 1:

A) Most highly enriched GO terms for the up-regulated mRNAs: Uninfected control vs Active Infection

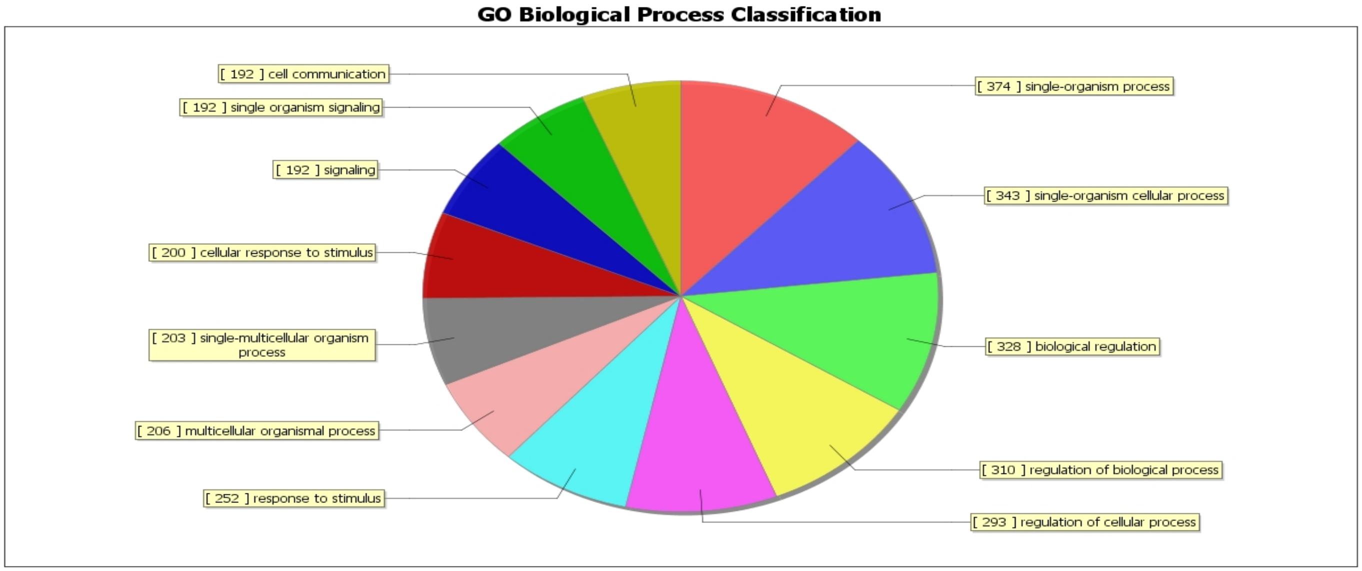

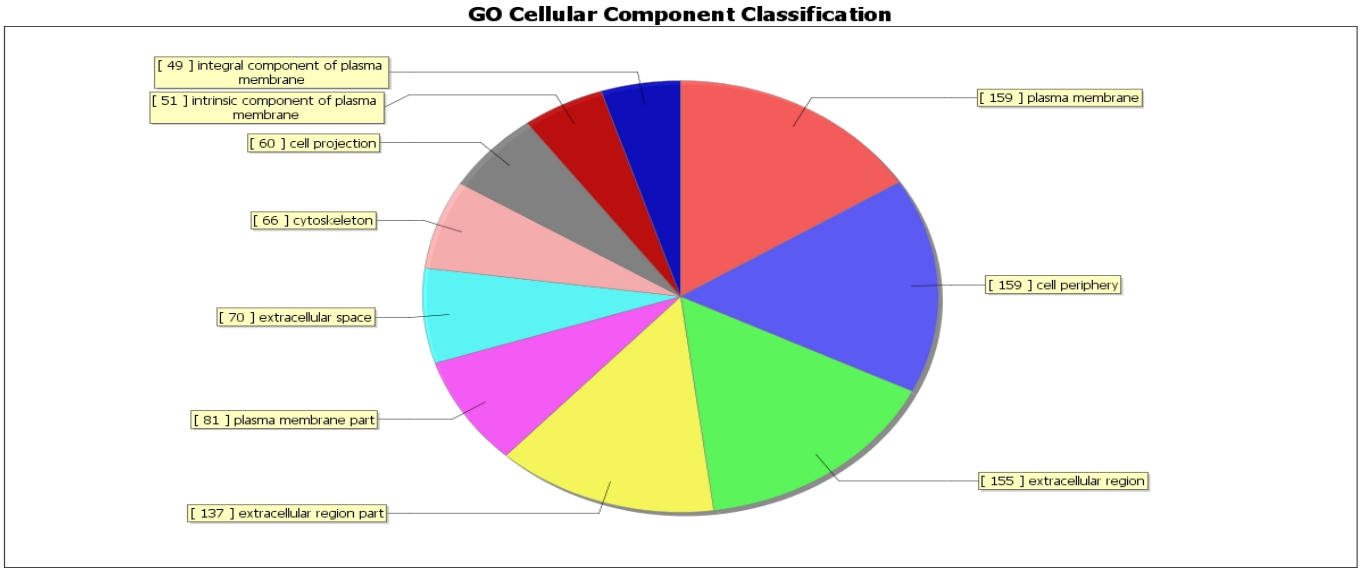

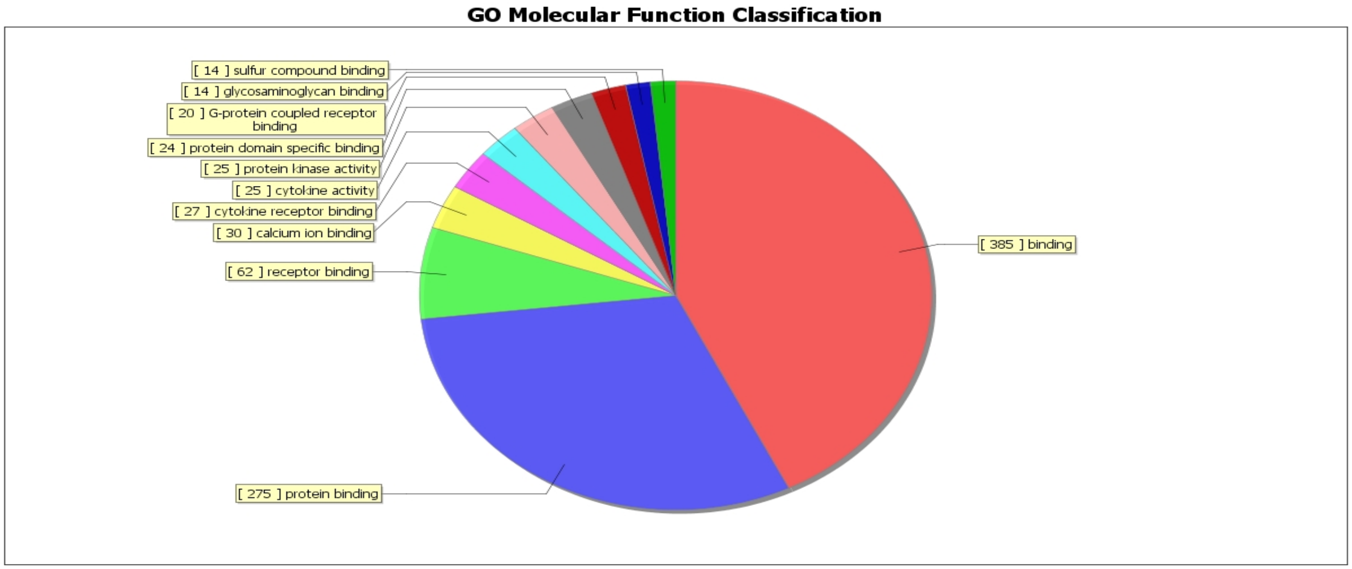

B) Most highly enriched GO terms for the down-regulated mRNAs: Uninfected control vs Active Infection

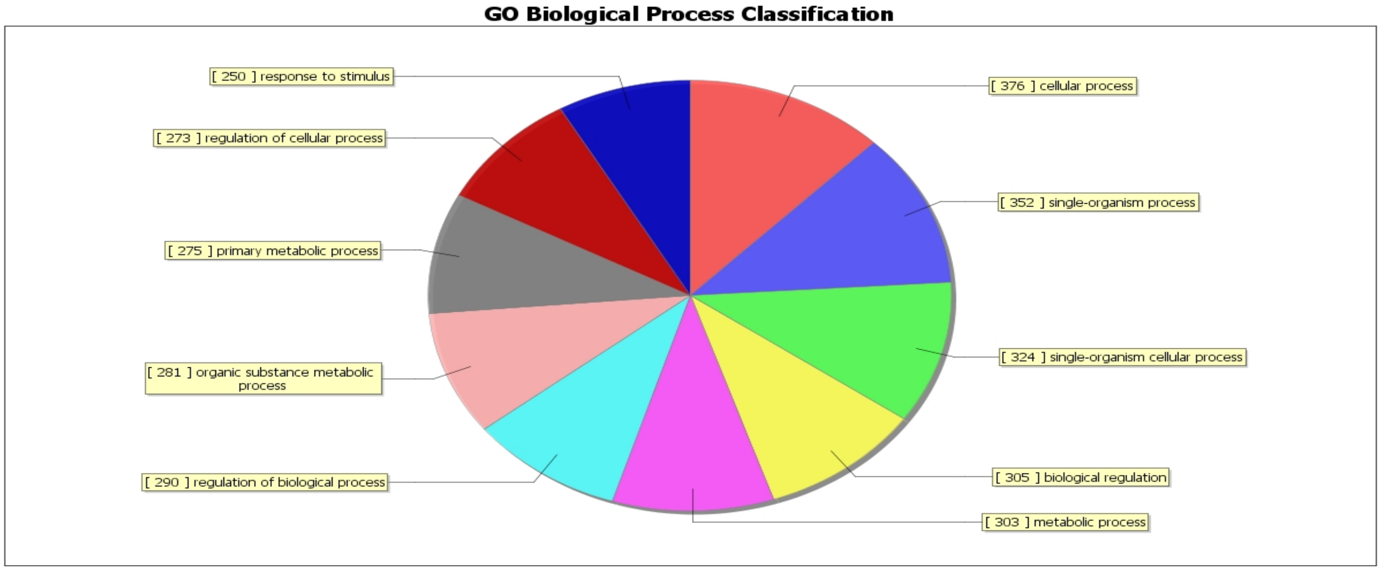

GO Cellular Component Classification

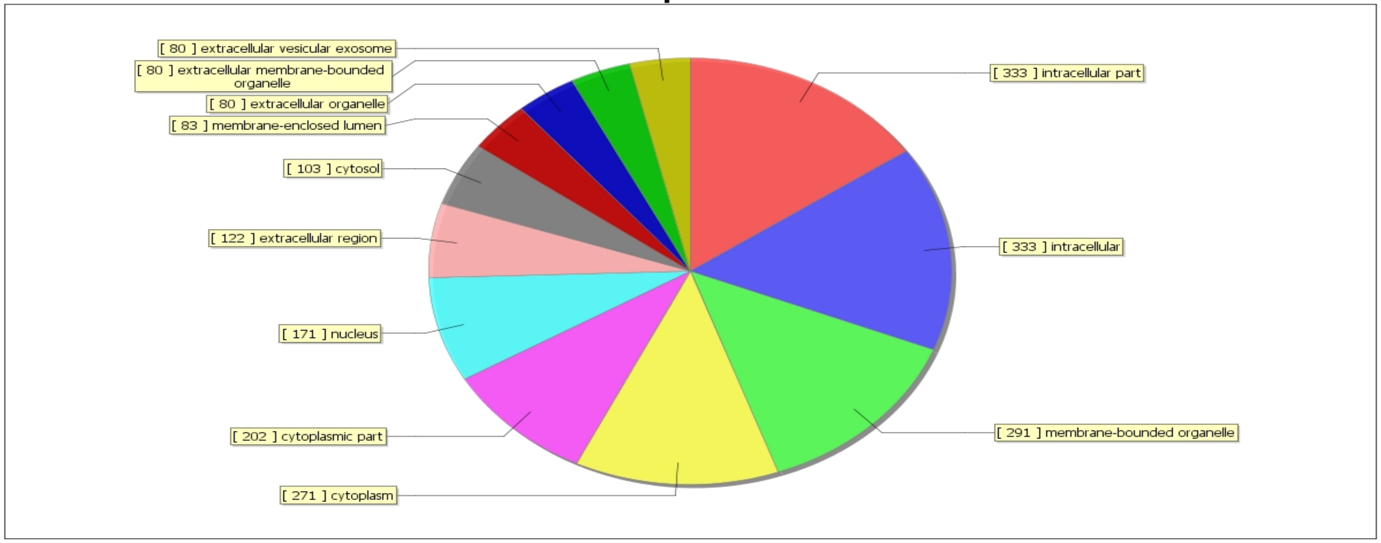

GO Molecular Function Classification

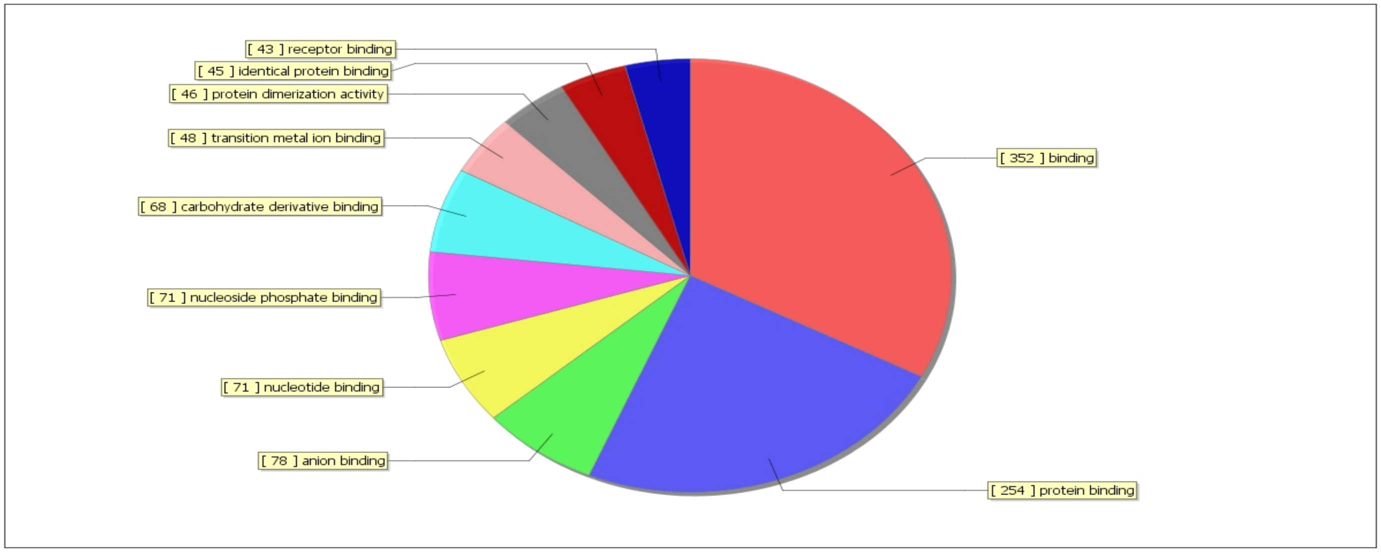

C) Most highly enriched GO terms for the up-regulated mRNAs: Uninfected control vs Latent Infection

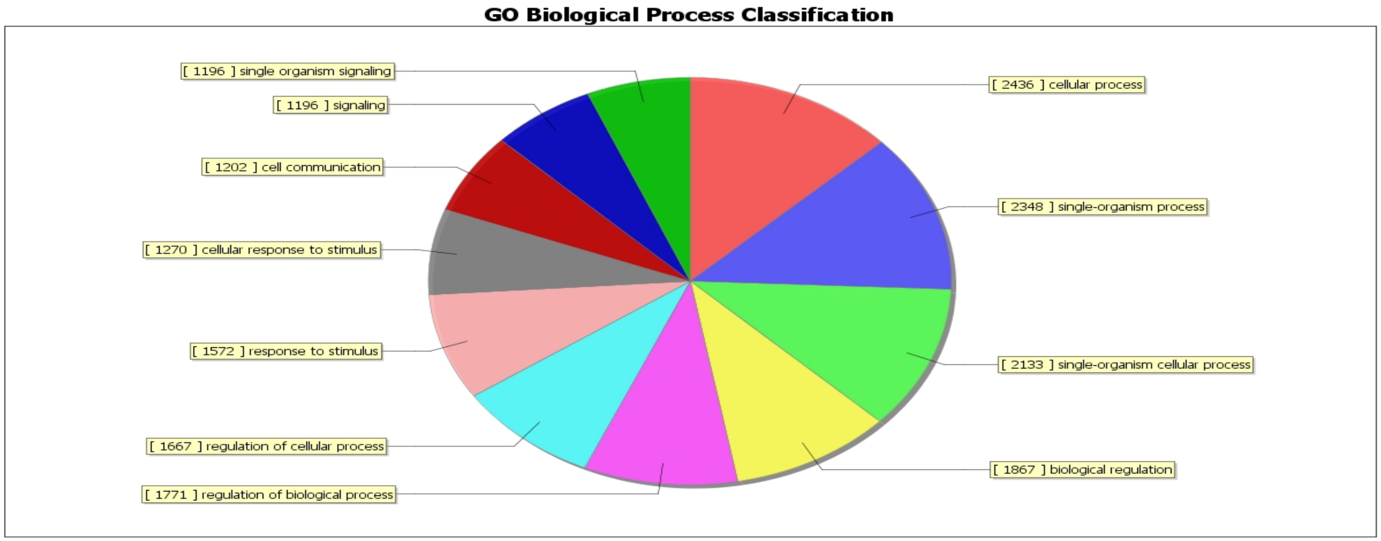

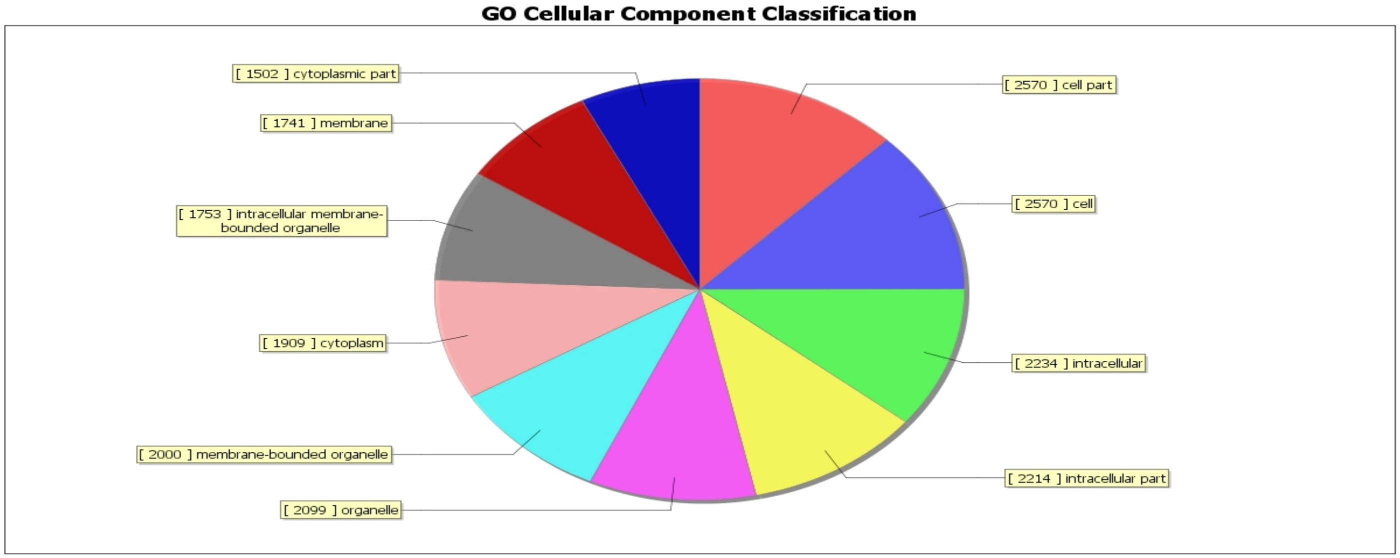

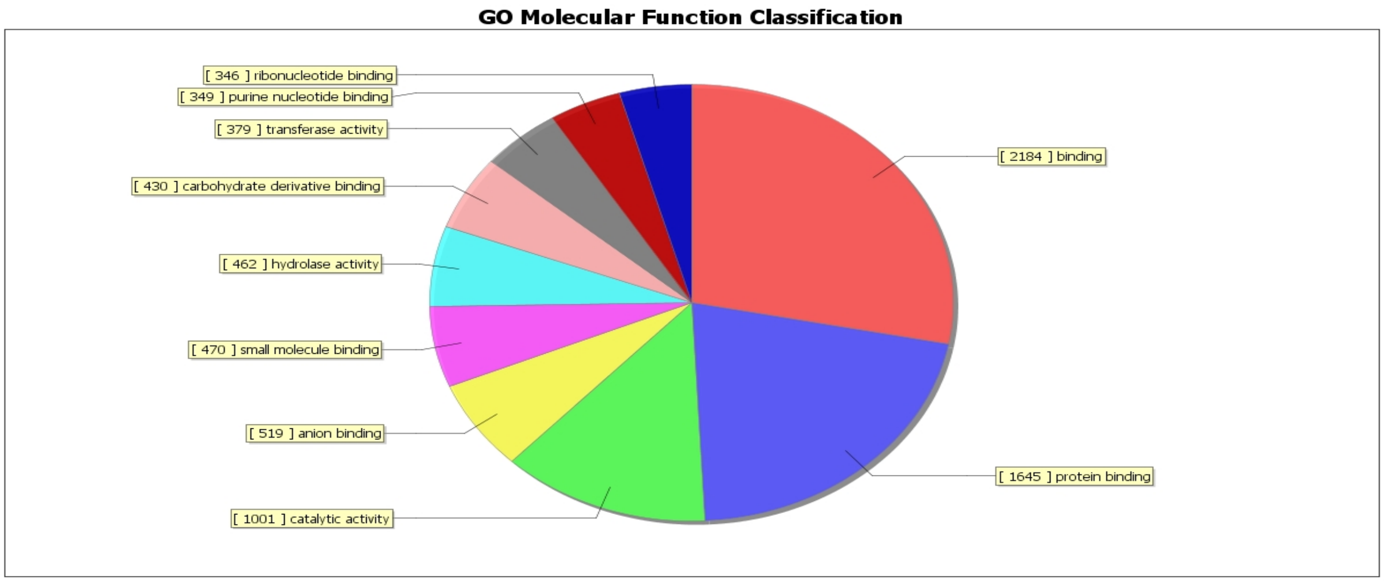

D) Most highly enriched GO terms for the Down-regulated mRNAs: Uninfected control vs Latent_Infection

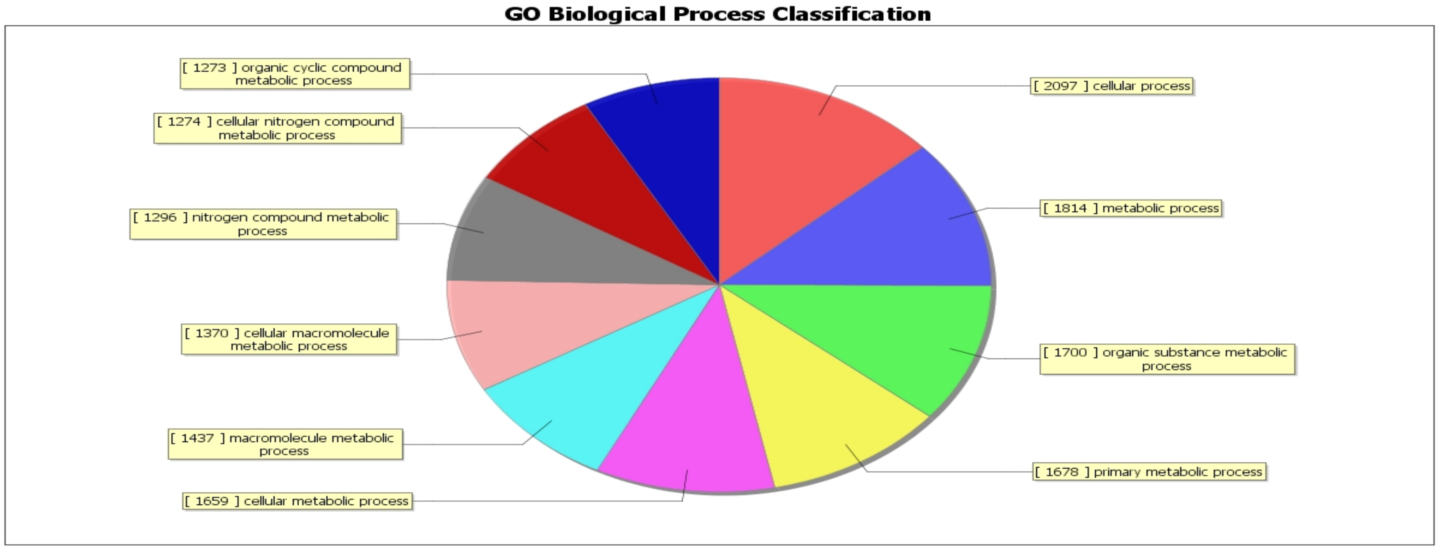

GO Cellular Component Classification

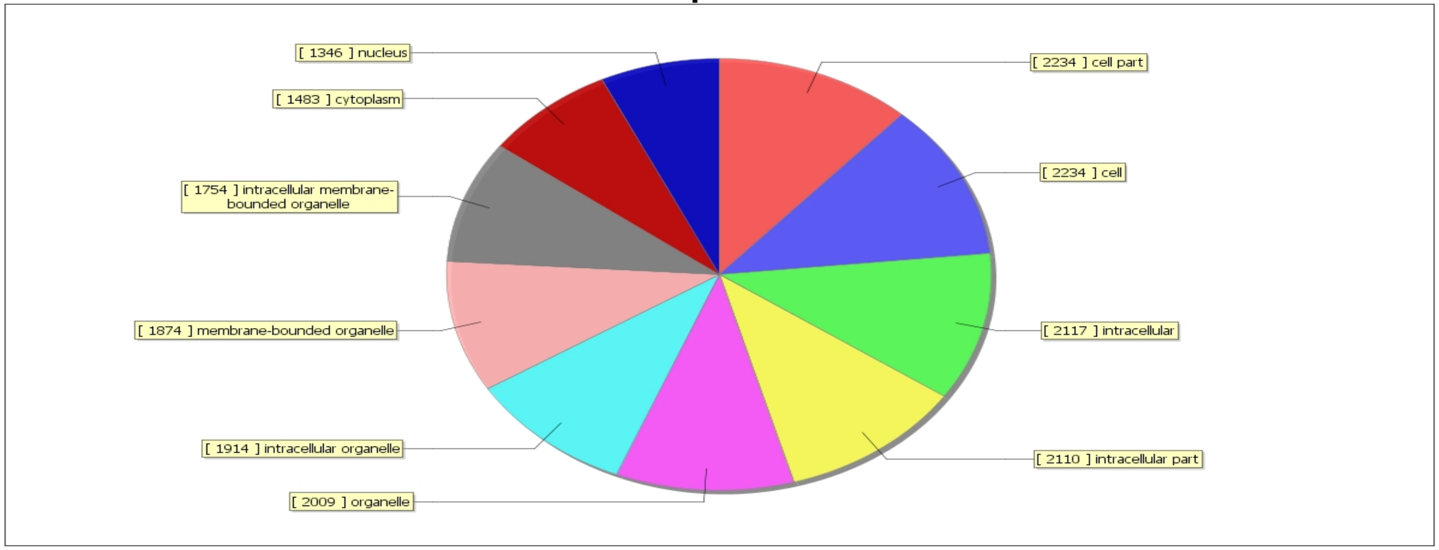

GO Molecular Function Classification

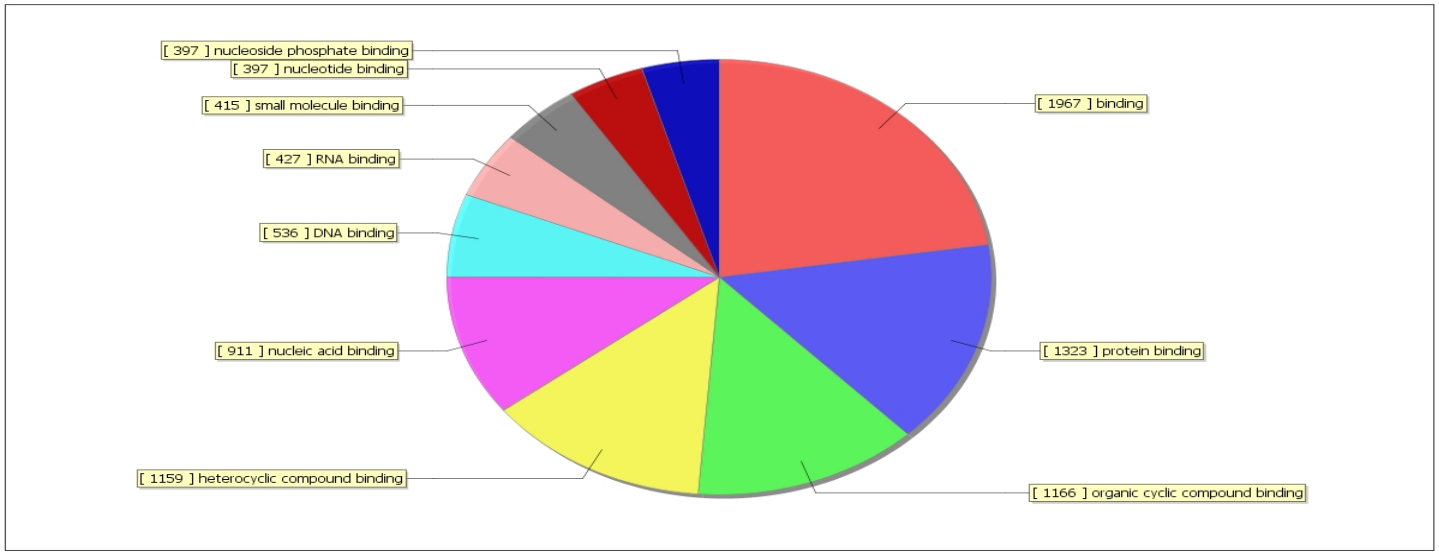

Supplementary Information:

Figure 7.

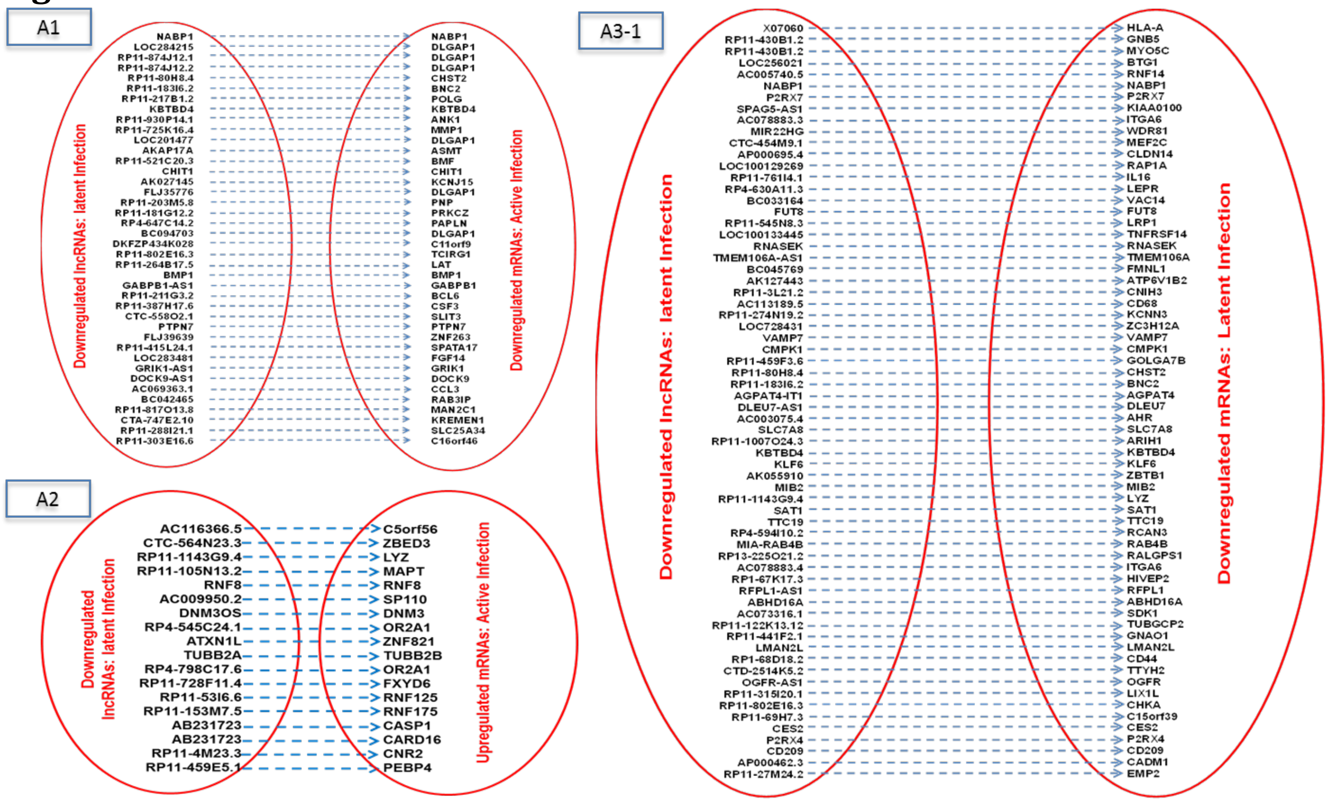

Figure 7 (contd.).

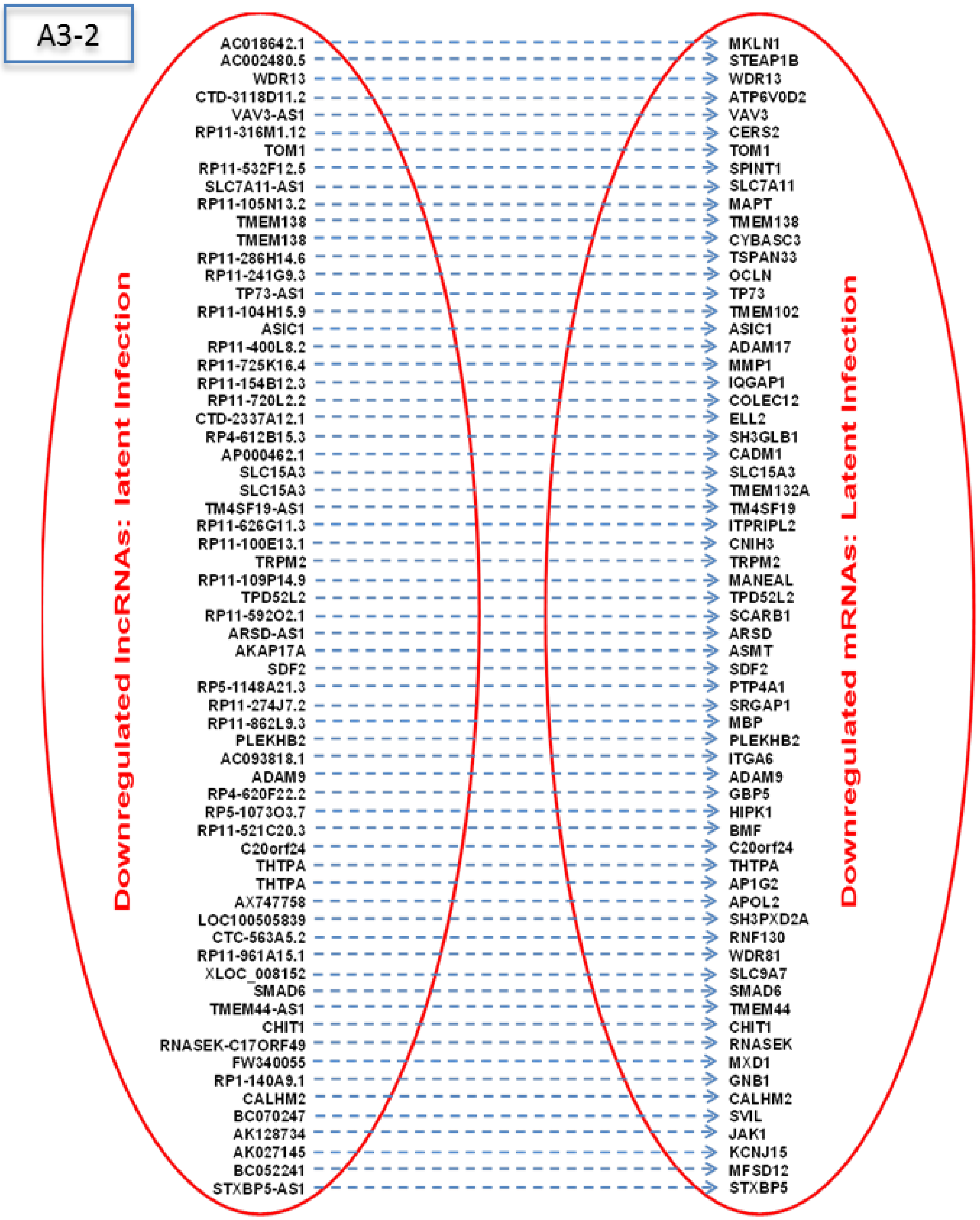

Figure 7 (contd.).

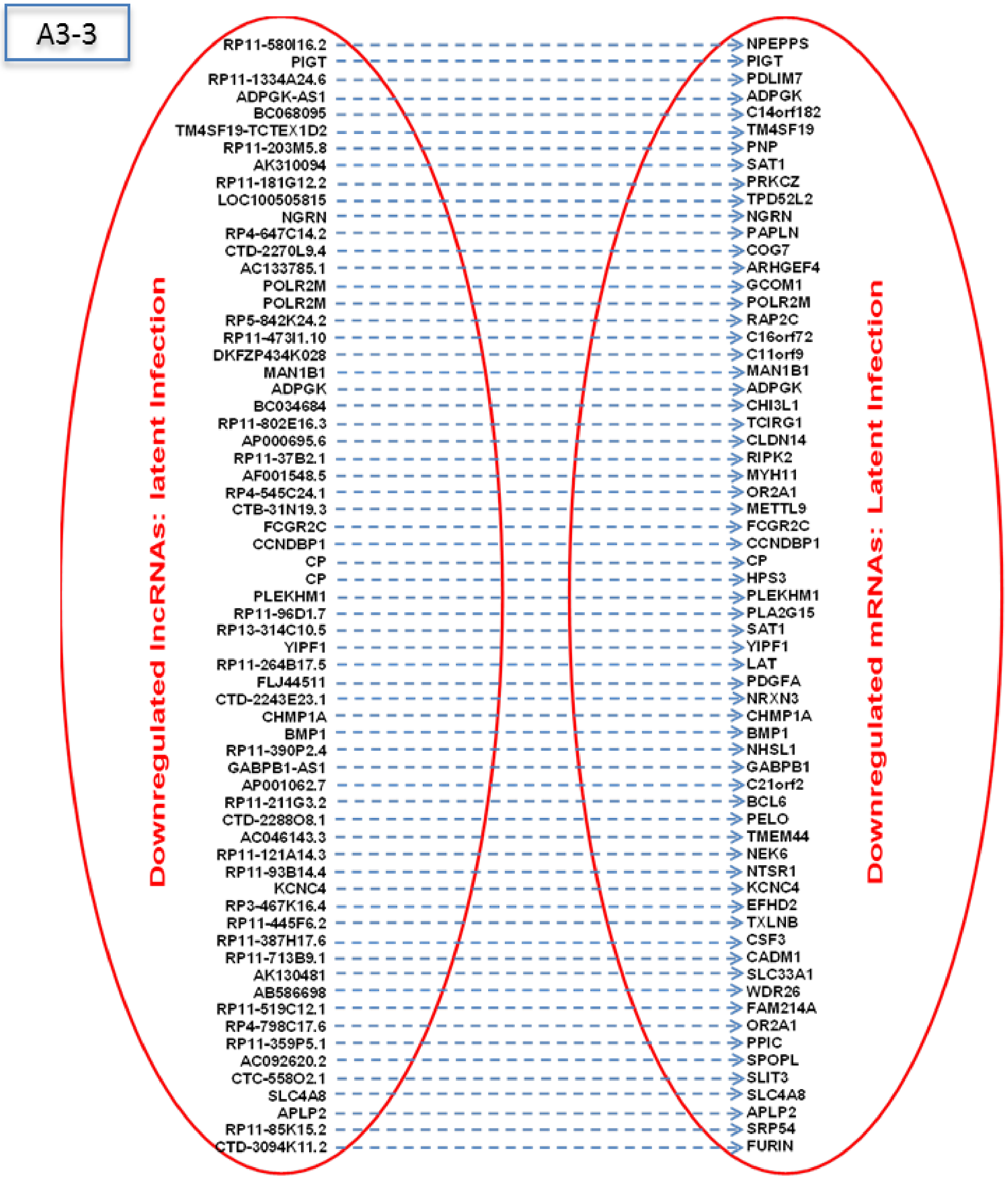

Figure 7 (contd.).

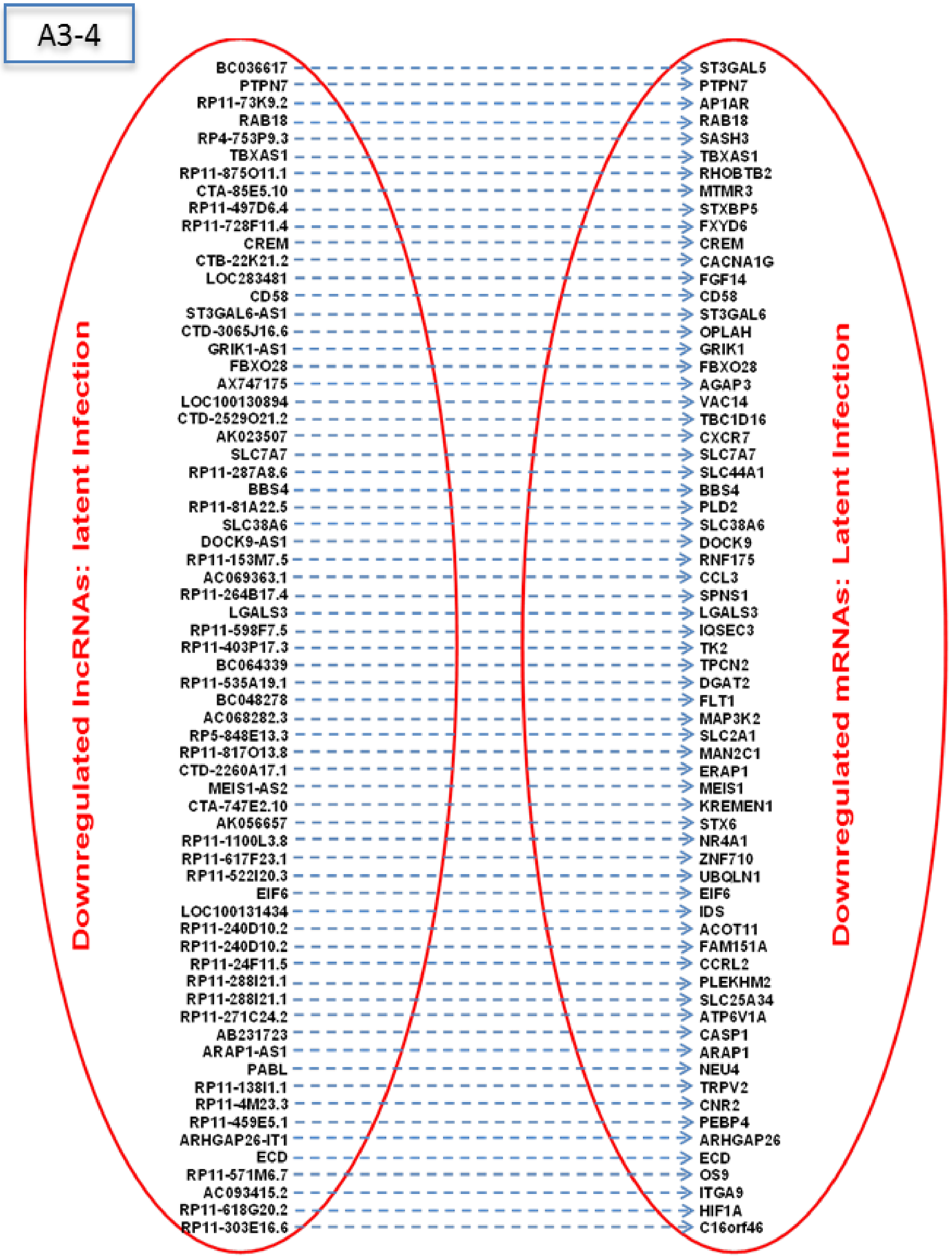

Figure 7 (contd.).

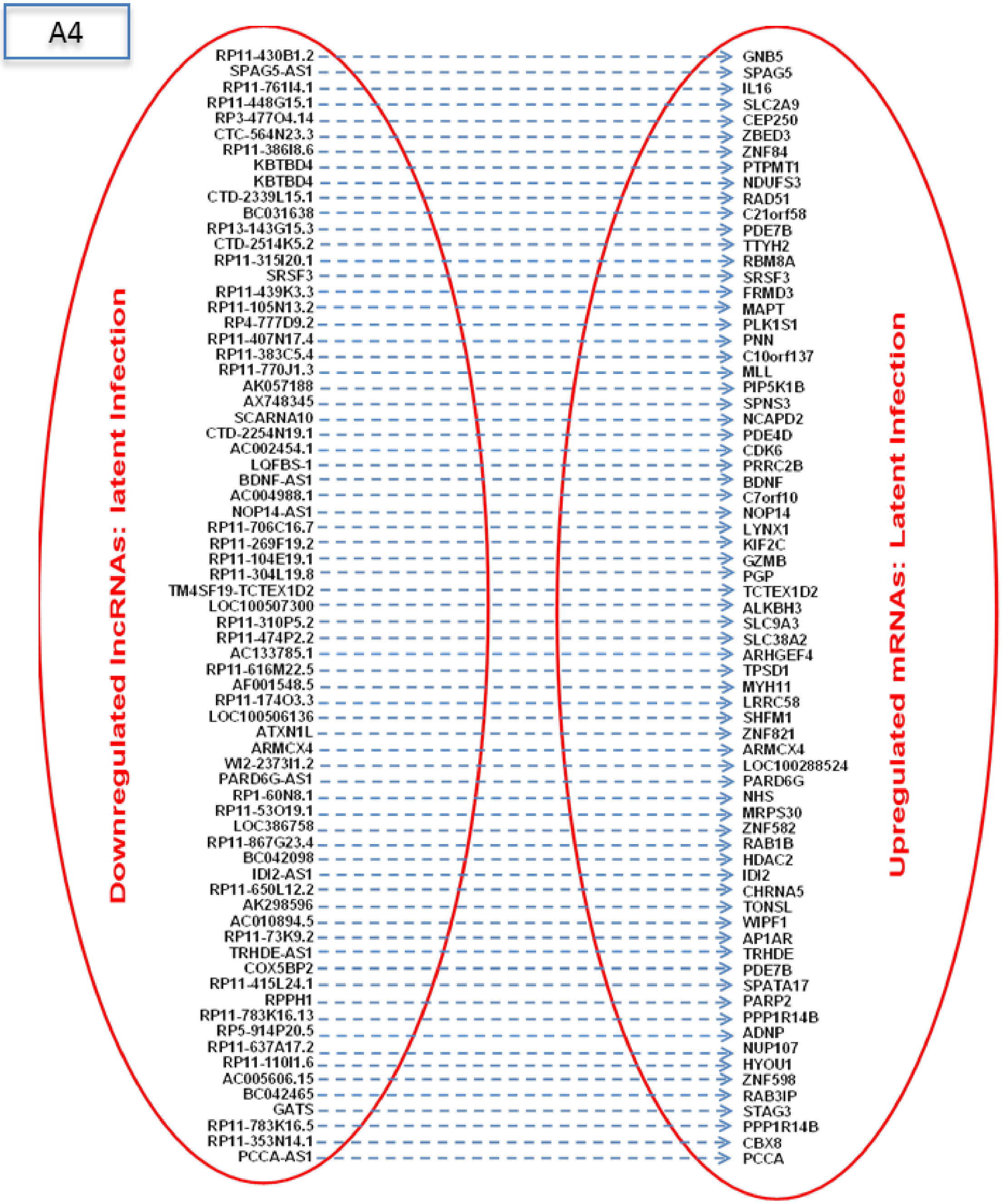

Figure 8.

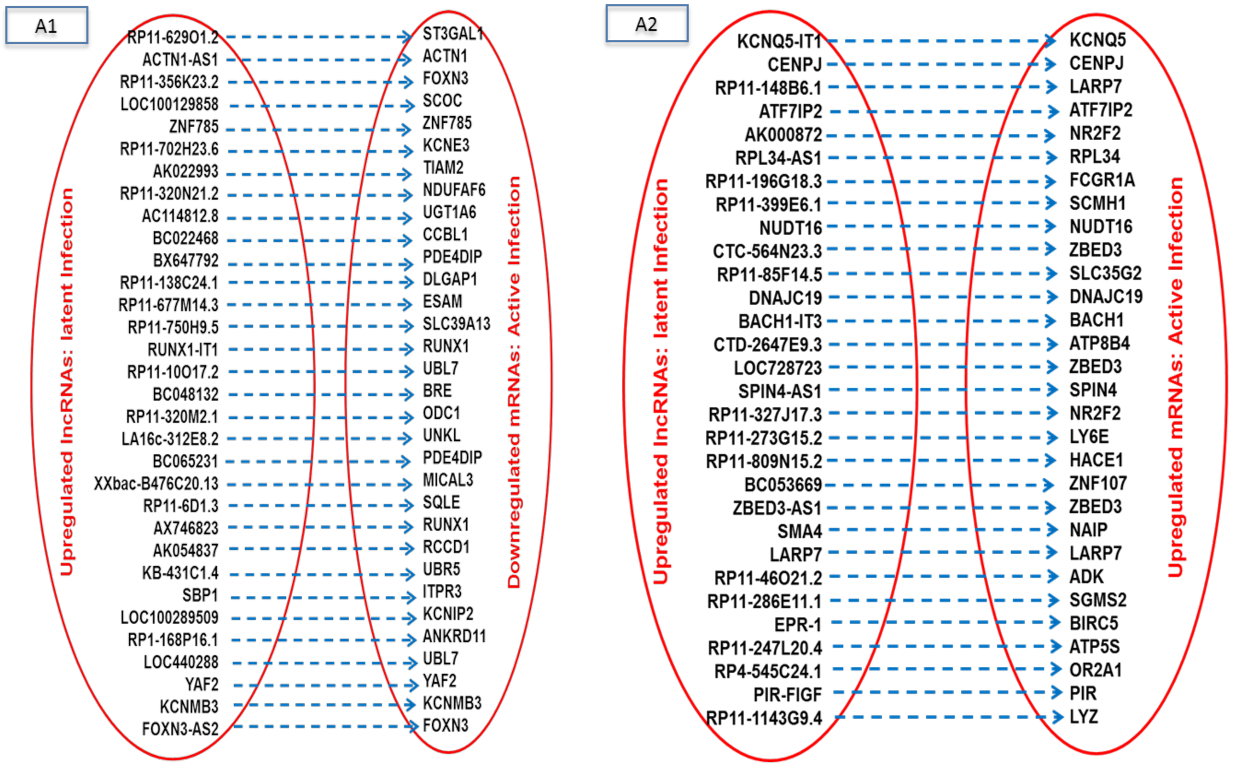
A1
Upregulated lncRNAs: latent Infection
Downregulated mRNAs: Active Infection
RP11-629O1.2
ACTN1-AS1
RP11-356K23.2
LOC100129858
ZNF785
RP11-702H23.6
AK022993
RP11-320N21.2
AC114812.8
BC022468
BX647792
RP11-138C24.1
RP11-677M14.3
RP11-750H9.5
RUNX1-IT1
RP11-10O17.2
BC048132
RP11-320M2.1
LA16c-312E8.2
BC065231
XXbac-B476C20.13
RP11-6D1.3
AX746823
AK054837
KB-431C1.4
SBP1
LOC100289509
RP1-168P16.1
LOC440288
YAF2
KCNMB3
FOXN3-AS2
ST3GAL1
ACTN1
FOXN3
SCOC
ZNF785
KCNE3
TIAM2
NDUFAF6
UGT1A6
CCBL1
PDE4DIP
DLGAP1
ESAM
SLC39A13
RUNX1
UBL7
BRE
ODC1
UNKL
PDE4DIP
MICAL3
SQLE
RUNX1
RCCD1
UBR5
ITPR3
KCNIP2
ANKRD11
UBL7
YAF2
KCNMB3
FOXN3
A2
Upregulated lncRNAs: latent Infection
Upregulated mRNAs: Active Infection
KCNQ5-IT1
CENPJ
RP11-148B6.1
ATF7IP2
AK000872
RPL34-AS1
RP11-196G18.3
RP11-399E6.1
NUDT16
CTC-564N23.3
RP11-85F14.5
DNAJC19
BACH1-IT3
CTD-2647E9.3
LOC728723
SPIN4-AS1
RP11-327J17.3
RP11-273G15.2
RP11-809N15.2
BC053669
ZBED3-AS1
SMA4
LARP7
RP11-46O21.2
RP11-286E11.1
EPR-1
RP11-247L20.4
RP4-545C24.1
PIR-FIGF
RP11-1143G9.4
KCNQ5
CENPJ
LARP7
ATF7IP2
NR2F2
RPL34
FCGR1A
SCMH1
NUDT16
ZBED3
SLC35G2
DNAJC19
BACH1
ATP8B4
ZBED3
SPIN4
NR2F2
LY6E
HACE1
ZNF107
ZBED3
NAIP
LARP7
ADK
SGMS2
BIRC5
ATP5S
OR2A1
PIR
LYZ

Figure 8 (contd.).

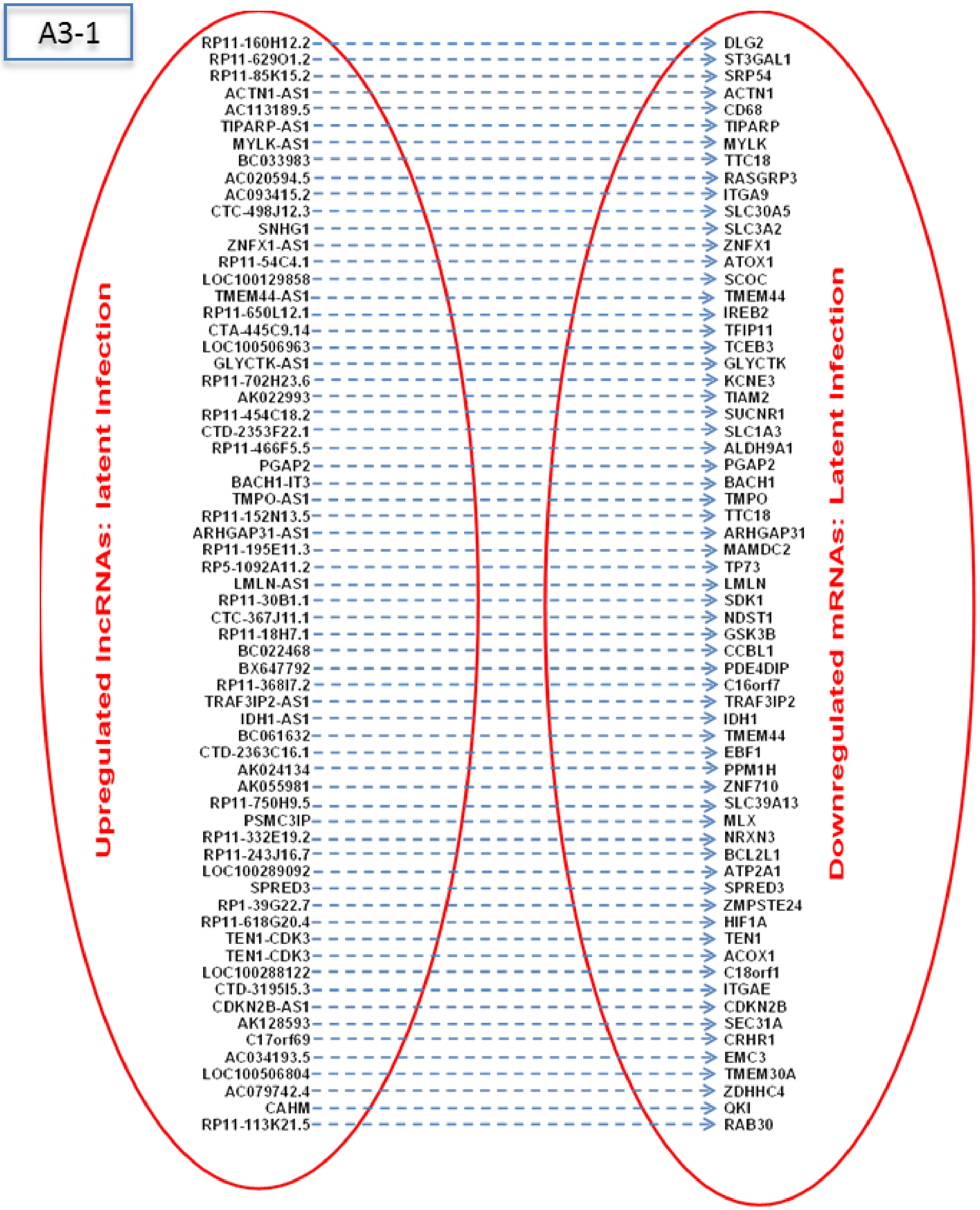

Figure 8 (contd.).

A3-2

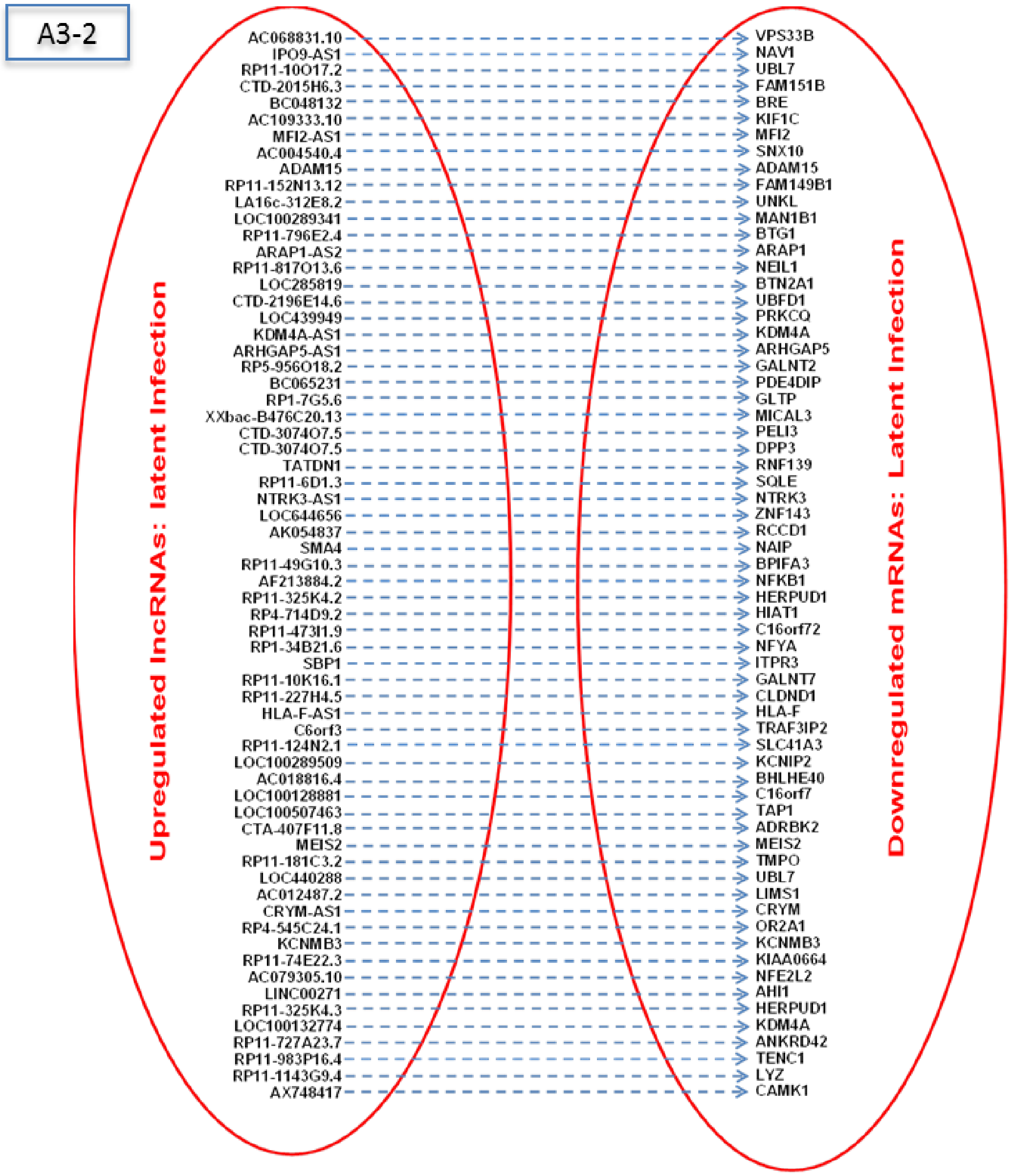

Figure 8 (contd.).

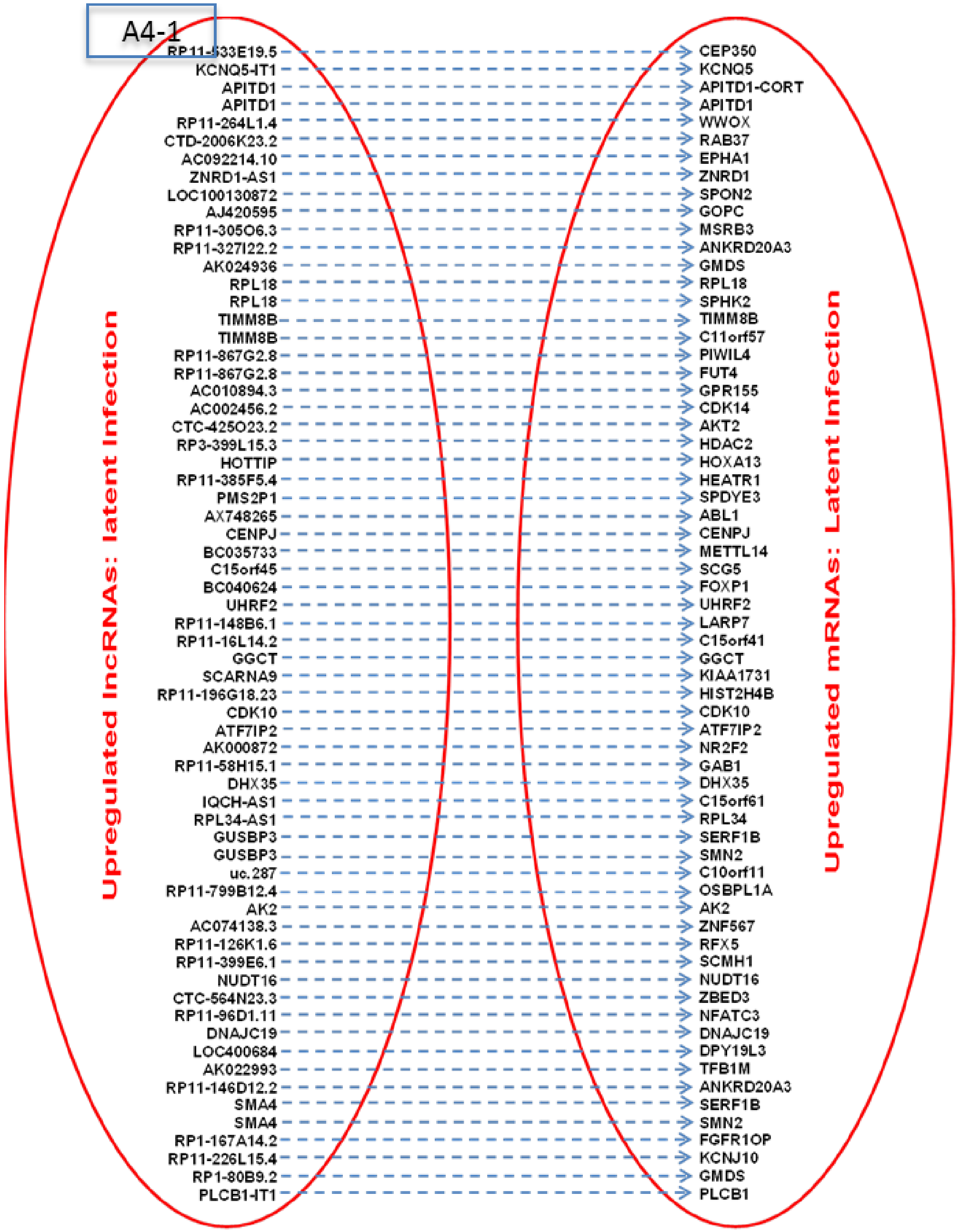

Figure 8 (contd.).

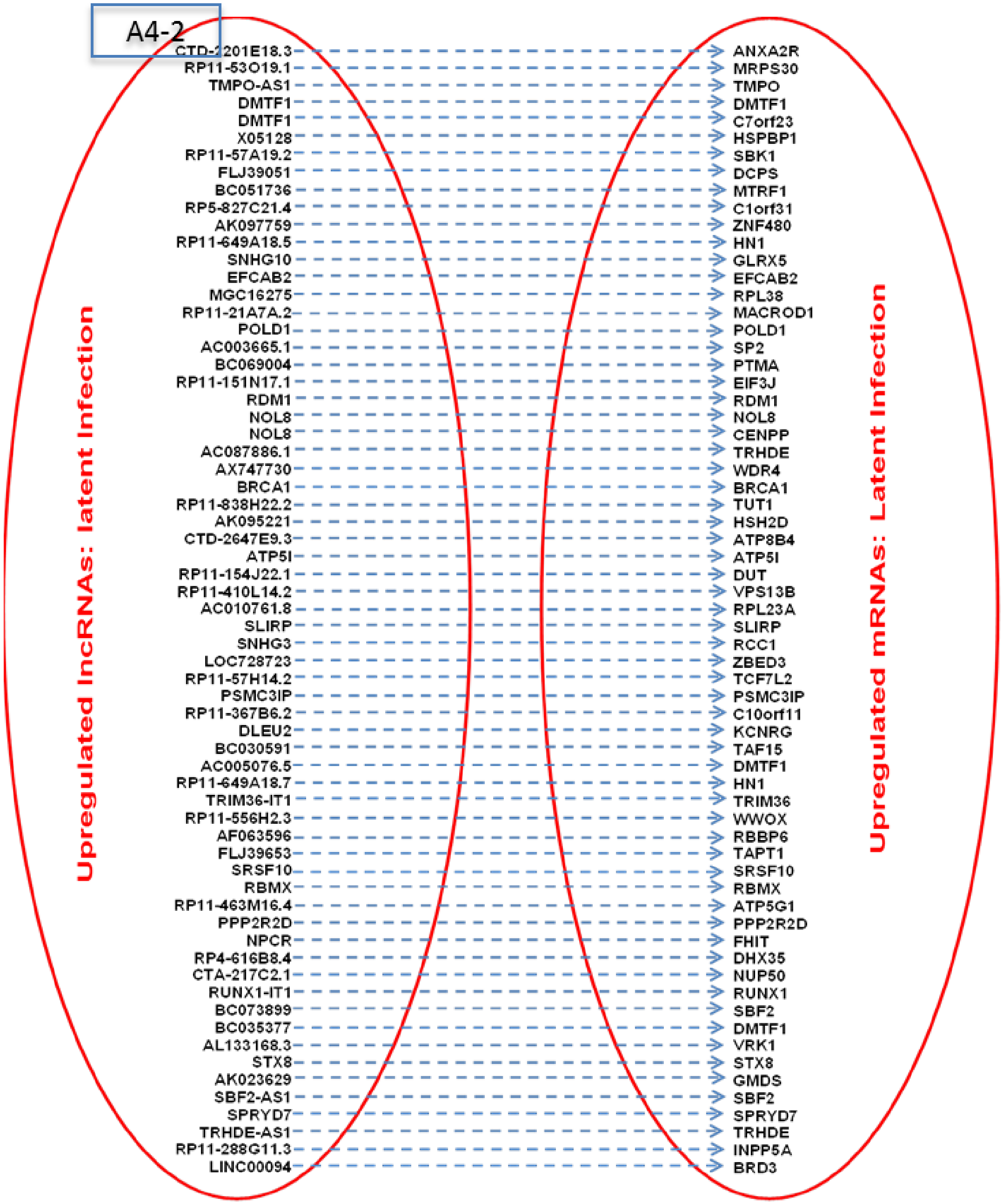

Figure 8 (contd.).

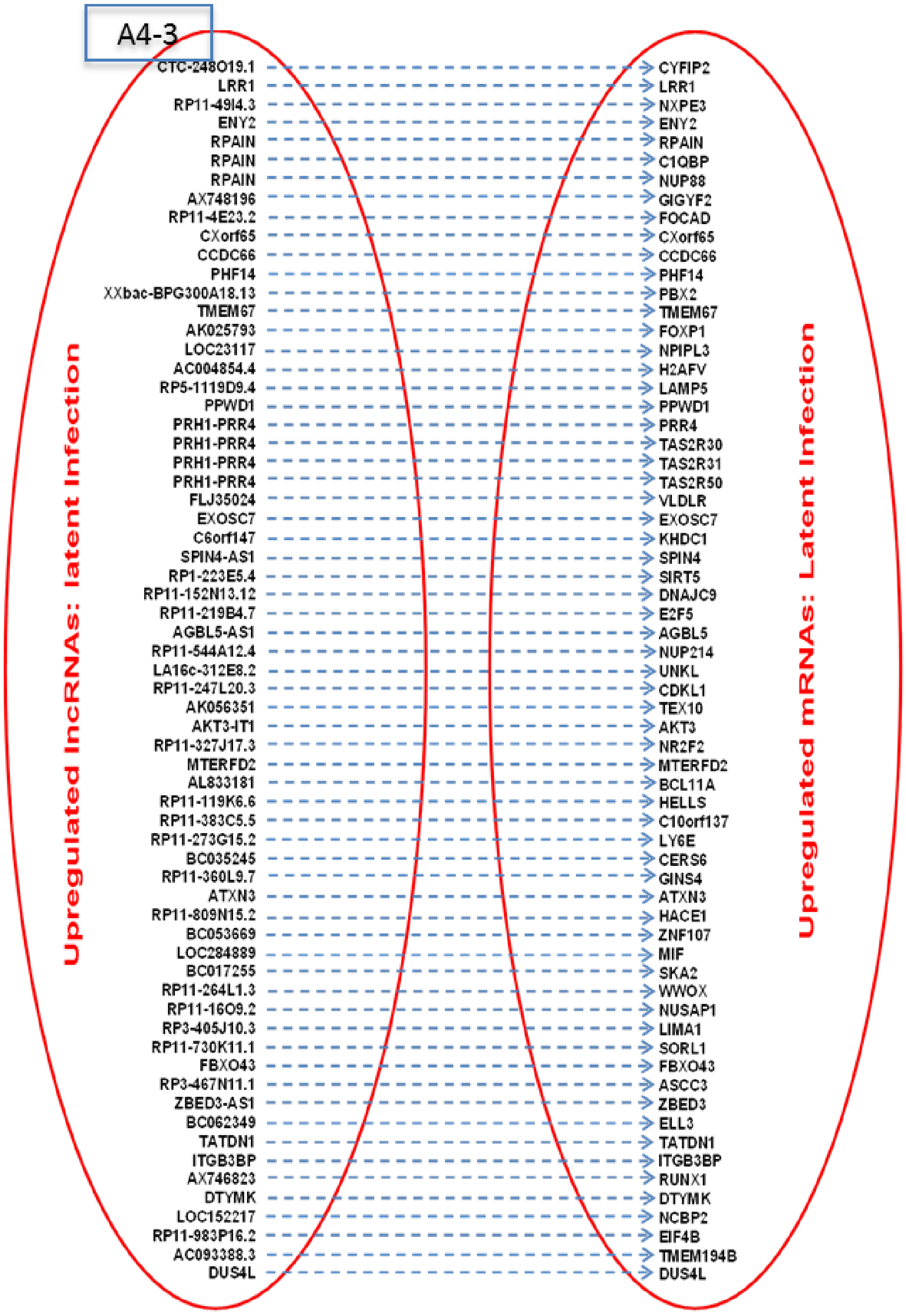

Figure 8 (contd.).

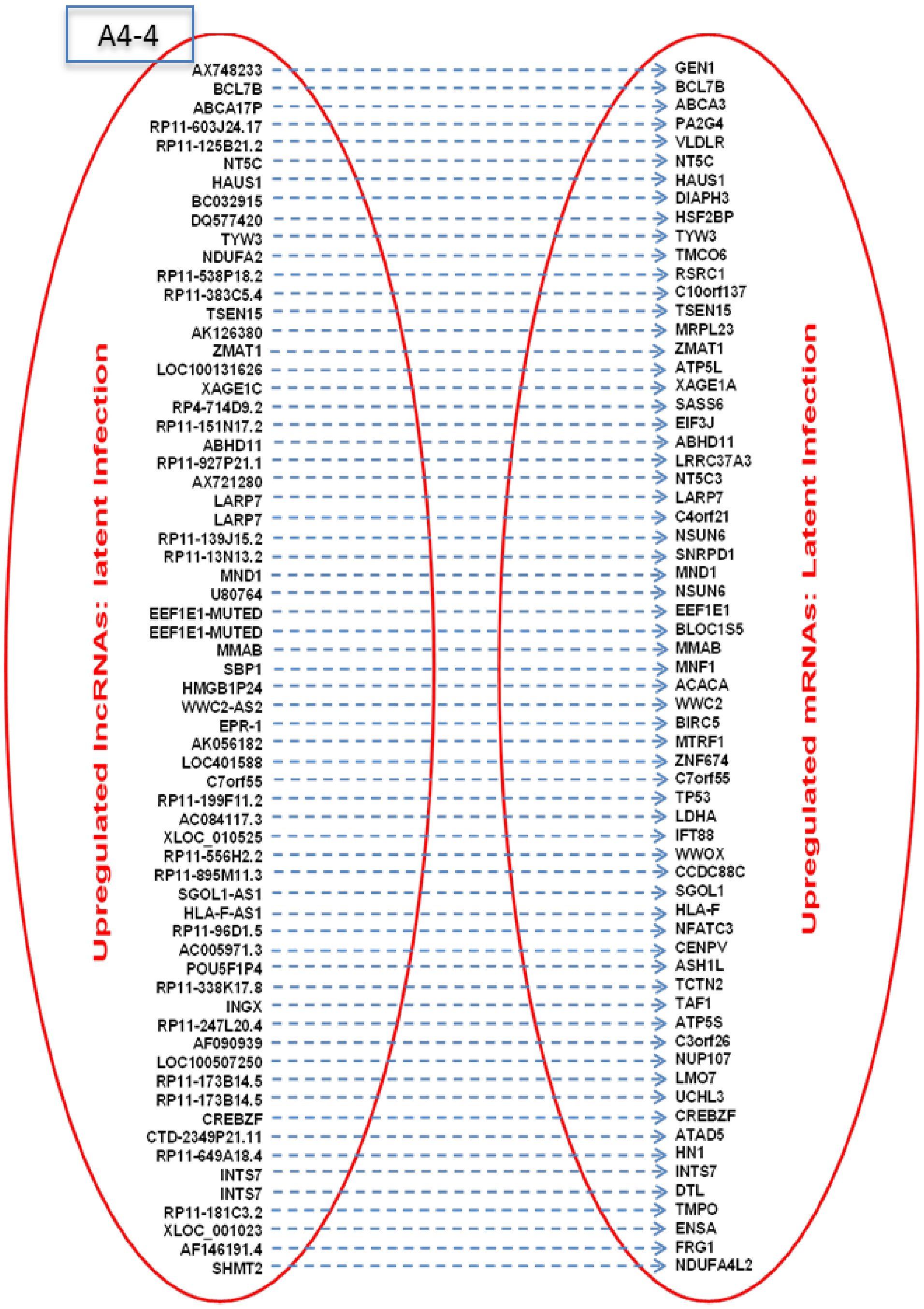

Figure 8 (contd.).

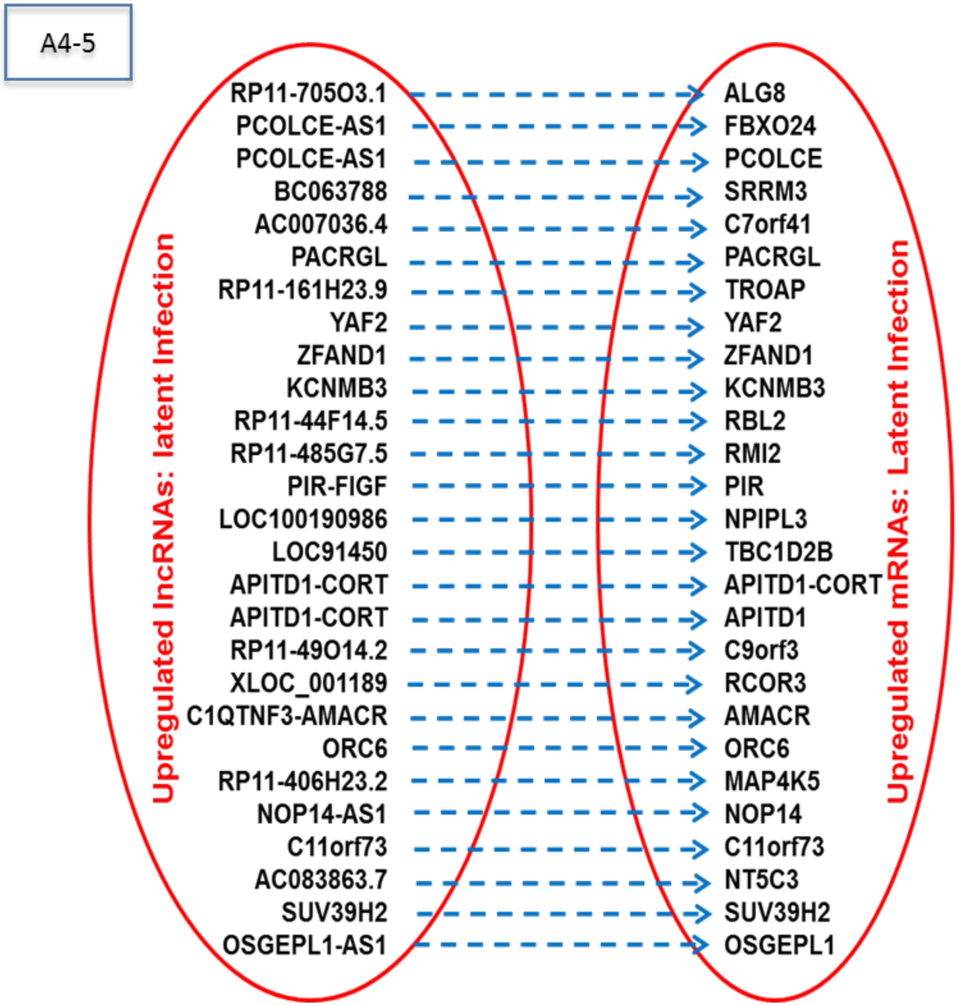

Supplement: Supplementary Information [file srep34862-s1.pdf]
